# Inhibition of TLR4 enhances oxaliplatin chemotherapy sensitivity in esophageal squamous cell carcinoma by suppressing inflammation and glycolysis

**DOI:** 10.1186/s12876-026-04663-2

**Published:** 2026-02-10

**Authors:** Ziqi Zhu, Meng Zhang, Zequn Di, Xin Tao, Yaohui Dai, Zhiqiang Zhan, Hongping Chen

**Affiliations:** 1 The MOE Basic Research and Innovation Center for the Targeted Therapeutics of Solid Tumors, School of Basic Medicine, Jiangxi Medical College, Nanchang University, Nanchang, Jiangxi China; 2 Jiangxi Provincial Key Laboratory of Tumor Biology, School of Basic Medicine, Jiangxi Medical College, Nanchang University, Nanchang, Jiangxi China; 3 Department of Oncology, Pingxiang People’s Hospital, No. 8 Wugong Mountain Avenue, Pingxiang, Jiangxi China; 4 School of Histology and Embryology, Jiangxi Medical College, Nanchang University, Nanchang, Jiangxi China

**Keywords:** Esophageal squamous cell carcinoma, Oxaliplatin, Toll-like receptor 4, Inflammation, Glycolysis

## Abstract

**Background:**

Oxaliplatin (OXA) has become a key chemotherapeutic agent in the treatment of esophageal squamous cell carcinoma (ESCC). Toll-like receptor 4 (TLR4) is frequently upregulated in OXA-treated tumors, yet its role in regulating OXA sensitivity in ESCC remains unclear. This study suggests that TLR4 acts as a critical regulator of OXA responsiveness through dual modulation of NF-κB p65-driven inflammation and HIF-1α/GLUT1-mediated glycolysis.

**Methods:**

Using ESCC cell lines and a 4-nitroquinoline 1-oxide (4-NQO)-induced murine ESCC model, we demonstrated that OXA upregulates TLR4 and its adaptor protein MYD88, thereby stimulating inflammatory cytokine production and activating glycolytic enzymes. Pharmacological inhibition or shRNA-mediated knockdown of TLR4 significantly enhanced the suppressive effects of OXA on ESCC proliferation, migration, and invasion in vitro.

**Results:**

TLR4 knockout markedly improved the efficacy of OXA in vivo, reducing tumor burden while simultaneously downregulating key inflammatory mediators and glycolytic markers.

**Conclusions:**

These findings indicate that TLR4 inhibition enhances OXA’s chemotherapeutic effects by attenuating both inflammation and glycolytic metabolism. Our results support TLR4 signaling as a pivotal modulator of OXA sensitivity in ESCC and propose TLR4 targeting as a promising strategy for improving OXA-based chemotherapy.

**Supplementary Information:**

The online version contains supplementary material available at 10.1186/s12876-026-04663-2.

## Introduction

Esophageal cancer is the seventh most prevalent malignancy and the sixth leading cause of cancer-related mortality worldwide, with a five-year survival rate below 30% due to frequent late-stage diagnosis and limited treatment efficacy [1, 2]. Esophageal squamous cell carcinoma (ESCC), the predominant histological subtype in East Asia and Africa, constitutes over 80% of global esophageal cancer cases [3]. In China, ESCC incidence is approximately 100-fold higher than in Western countries [1, 4], with risk factors including smoking, alcohol consumption, and nutritional deficiencies [5]. Despite advances in multimodal therapies, more than 50% of ESCC patients present with unresectable or metastatic disease at diagnosis [6], highlighting the urgent need for strategies to improve therapeutic sensitivity.

Oxaliplatin (OXA), a platinum-based chemotherapeutic agent widely used in gastrointestinal cancers [7, 8], has shown promise in ESCC treatment [9, 10]. However, its efficacy is often limited by intrinsic and adaptive resistance mechanisms [11, 12], including DNA damage repair, pro-survival signaling pathways, and metabolic reprogramming [13, 14]. Toll-like receptor 4 (TLR4) serves as a critical mediator of tumor adaptation to therapy [15, 16]. Notably, TLR4 activation confers resistance to anti-EGFR agents in head and neck squamous cell carcinoma [17]. Yet, its role varies across cancer types [18, 19]. For instance, TLR4 promotes hepatocellular carcinoma metastasis via the ADAM10/CX3CL1 axis [20], while in non-small cell lung cancer, it suppresses progression through NLRP3/GSDMD-dependent pyroptosis [21].

Elevated TLR4 expression correlates with aggressive tumor behavior and poor prognosis in breast cancer and colorectal cancer [22] and has also been documented in ESCC [23, 24]. In peripheral nerves, OXA-induced TLR4 upregulation contributes to chemotherapy-induced neuropathy [25], and our prior work demonstrated that COX-2 inhibition alleviates OXA-associated neuropathic pain [26]. These findings prompted us to investigate whether TLR4 inhibition could enhance OXA sensitivity in ESCC.

Growing evidence implicates TLR4 in macrophage polarization and glycolytic reprogramming [27, 28], a metabolic hallmark of cancer that sustains tumor growth via upregulation of glucose transporters (e.g., GLUT1) and glycolytic enzymes (e.g., PFKM, LDHB) [29]. TLR4 activation fuels tumor progression by modulating both inflammatory and metabolic pathways. For example, in hepatocellular carcinoma, TLR4 enhances glycolysis through HIF-1α/GLUT1 signaling, thereby reducing therapeutic vulnerability [28, 30]. Whether this axis operates in ESCC remains unknown. HIF-1α, a master metabolic regulator, drives the expression of glucose transporters and glycolytic enzymes [31], and GLUT1 overexpression is strongly associated with tumor aggressiveness and poor prognosis in multiple malignancies [32–34], including ESCC [35, 36]. Given that metabolic reprogramming may compromise OXA efficacy, understanding these mechanisms is critical.

This study aims to elucidate the role of TLR4 signaling in OXA chemotherapy and explore its underlying mechanisms. Our findings may provide novel insights to improve therapeutic strategies for esophageal cancer.

## Materials and methods

### 4-NQO-induced esophageal cancer mouse model

Male Toll-like receptor 4 knockout (TLR4^−/−^, C57BL/10ScN background) and wild-type (WT, C57BL/10ScN background) mice were obtained from the Animal Model Institution of Nanjing University, China. Mice were anesthetized using isoflurane (induction: 4%, maintenance: 1.5-2%) delivered in oxygen via a precision vaporizer. The depth of anesthesia was confirmed by the absence of a pedal withdrawal reflex and no response to a tail clamp. At the experimental endpoints, euthanasia was performed via cervical dislocation while the animals were under sustained surgical-plane anesthesia. This method was employed in accordance with the AVMA Guidelines for the Euthanasia of Animals to ensure a rapid and painless death following the loss of consciousness induced by anesthesia. All animal experimental procedures were conducted in accordance with the ethical standards and approval of the Nanchang University Animal Care Committee of the Medical College (Approval No. NCULAE-20220624037) and adhered to the principles outlined in the ARRIVE guidelines, ensuring animal welfare and compliance throughout the study. Six-week-old male mice were randomly assigned to four experimental groups (*n* = 7 per group): (1) WT control group (WT group); (2) WT mice treated with 4-nitroquinoline 1-oxide (4-NQO) and oxaliplatin (OXA) (WT + 4-NQO + OXA group); (3) TLR4 knockout mice group (TLR4^−/−^ group); and (4) TLR4 knockout mice treated with 4-NQO and OXA (TLR4^−/−^ + 4-NQO + OXA group). Mice received drinking water containing 100 µg/mL 4-NQO (N8141, Sigma) ad libitum for 10 weeks. Subsequently, OXA (5 mg/kg) was administered via intraperitoneal injection once weekly during weeks 11–14 and 17–19. At week 20 (one week after the final OXA injection), mice were anesthetized and euthanized. Body weight was monitored weekly. Serum and esophageal tissues were collected for further analysis.

### Cell culture

Human esophageal squamous cell carcinoma (ESCC) cell lines, ECA-109 (h056, iCell), KYSE-410 (CL-0586, Procell), KYSE-510 (CL-0737, Procell), and TE-1 (CL-0231, Procell), were authenticated by short tandem repeat (STR) profiling and maintained in RPMI-1640 medium (31880, Solarbio) supplemented with 10% fetal bovine serum (FBS; C04001-500, VivaCell). The normal human esophageal epithelial cell line HET-1 A (iCell-h333, iCell) was cultured in specialized medium (CC-3170, iCell) containing 10% FBS. All cells were incubated at 37 °C in a humidified 5% CO₂ atmosphere. Based on preliminary cell viability assays (Fig. S1), the following inhibitors were applied for 24 h: TAK-242 (TLR4 inhibitor, 100 nM, MedChemExpress) and ST2825 (MYD88 inhibitor, 3 µM, MedChemExpress).

### Lentiviral transfection and pharmacological inhibition

TLR4-targeting shRNA lentiviral plasmids (GenePharma, Jiangsu; sh*TLR4*) and MYD88-targeting shRNA lentiviral plasmids (OBiO, Shanghai; sh*MYD88*), along with their respective control vectors (Vector), were transfected into ECA-109 cells following the manufacturer’s protocol. Transfected cells were selected using puromycin (5 µg/mL) for 7 days, and knockdown efficiency was confirmed via Western blot and qRT-PCR (Fig. S2).

### Western blot

Total proteins were extracted using RIPA lysis buffer (R0010, Solarbio), and protein concentration was determined via BCA assay (P0009, Beyotime). Equal amounts of protein were separated by SDS-PAGE and transferred to a polyvinylidene difluoride (PVDF) membrane (Millipore, Cat. No. 24937-79-9). The membranes were probed with the following primary antibodies: MYD88 (1:250, YM33092, Immunoway), COX-2 (1:400, YM8171, Immunoway), TLR4 (1:100, AF2006, Affinity), and β-actin (1:1000, AF7018, Affinity). After incubation with HRP-conjugated secondary antibody (1:20,000, Immunoway), protein bands were visualized using an enhanced chemiluminescence (ECL) detection kit (Vazyme Biotechnology, Nanjing, China). Band intensities were quantified using ImageJ software (National Institutes of Health, Bethesda, MD, USA), and relative protein expression levels were normalized to β-actin.

### Quantitative Real-Time PCR (qRT-PCR)

Total RNA was isolated using TRIzol Reagent (15596-026, Thermo Fisher Scientific, Waltham, MA, USA) following the manufacturer’s protocol. RNA concentration and purity were assessed using a NanoDrop spectrophotometer (NanoDrop Technologies, Wilmington, DE, USA). First-strand cDNA was synthesized from 1 µg of total RNA using Hifair III 1st Strand cDNA Synthesis SuperMix (Yeasen Biotechnology, Shanghai, China). qPCR amplification was performed with SYBR Green Master Mix (11203ES08, Yeasen) under the following cycling conditions: initial denaturation, 95 °C for 5 min; amplification (40 cycles): 95 °C for 10 s, 60 °C for 30 s. β-actin was used as the endogenous control for normalization. Relative gene expression levels were calculated using the 2^−ΔΔCt^ method (each with 3 technical replicates). Primer sequences are provided in Supplementary Table S1.

### Immunohistochemistry (IHC), Immunofluorescence (IF), and Immunocytochemistry (ICC) Immunocytochemistry (ICC)

For ICC, cells were seeded onto 24-well chamber slides and fixed with 4% paraformaldehyde. For IHC and IF, 5-µm paraffin-embedded esophageal tissue sections were subjected to antigen retrieval in citrate buffer (pH 6.0) and blocked with 3% H_2_O_2_ to quench endogenous peroxidase activity. All samples were incubated overnight at 4 °C with the following primary antibodies: MYD88 (1:250, YM33092, Immunoway), COX-2 (1:400, AF7003, Affinity), Cyclin D1 (1:400, ab16663, Abcam), S100A8 (1:250, DF6556, Affinity), S100A9 (1:250, DF7596, Affinity) and PCNA (1:500, AF0239, Affinity). After PBS washes, samples were incubated for 2 h at room temperature with: horseradish peroxidase (HRP)-conjugated secondary antibody (ZSGB-BIO, Beijing, China) for IHC and ICC; and fluorophore-conjugated secondary antibody (1:1000, RS0001/RS0002/RS23620, Immunoway) for IF. Nuclear counterstaining was performed using: crystal violet for IHC and ICC; and DAPI (4’,6-diamidino-2-phenylindole) for IF.

### Hematoxylin and Eosin (H&E) staining

Tissue sections were stained using a Hematoxylin-Eosin (H&E) Stain Kit (G1120, SolarBio Science) following the manufacturer’s protocol.

### MTT cell viability assay

Cells were seeded at a density of 4 × 10³ cells/well in 96-well plates. After 24-hour treatments, cells were incubated with MTT solution (5 µg/mL) for 3 h at 37 °C. The formazan crystals were dissolved in 150 µL DMSO per well, and absorbance was measured at 490 nm using a microplate reader (each with 6 technical replicates).

### Colony formation assay

Cells were plated at 600 cells/well in 6-well plates and cultured for 14 days. Colonies were fixed with 4% paraformaldehyde and stained with 1% Giemsa solution. Only colonies containing over 50 cells were counted manually under a light microscope.

### Migration and invasion assays

#### Migration assay

Cells (5 × 10³) suspended in serum-free medium were seeded into the upper chamber of Transwell inserts (8 μm pores; 3422, Corning). The lower chamber was filled with 750 µL of RPMI-1640 medium supplemented with 20% FBS as a chemoattractant. After 24 h of incubation at 37 °C in 5% CO₂.

#### Invasion assay

Transwell membranes were pre-coated with Matrigel^®^ matrix (40183, Yeasen) and allowed to polymerize at 37 °C for 1–2 h. Cells (5 × 10³) in serum-free medium were then seeded onto the coated membranes.

For both migration and invasion assays, non-migratory/non-invasive cells remaining on the upper membrane surface were gently removed using sterile cotton swabs, after which the transmigrated cells were fixed with 4% paraformaldehyde (PFA) for 15 min at room temperature, stained with 1% Giemsa solution for 20 min, and subsequently imaged using an inverted phase-contrast microscope. Quantitative analysis was performed by either manual counting of five random fields per membrane or automated analysis using ImageJ software.

### Enzyme-Linked Immunosorbent Assay (ELISA)

Serum concentrations of IL-6 (JL20268, Jonin) and IL-1β (KE10003, Proteintech) were quantified using commercial ELISA kits according to manufacturers’ protocols. Briefly, standards and samples were added to antibody-precoated wells, followed by sequential incubations with detection antibodies, enzyme conjugates, and substrate solutions. Absorbance was measured at the specified wavelengths using a microplate reader, and cytokine concentrations were determined by extrapolation from standard curves.

### Lactate production assay

Cells were plated in 6 cm culture dishes and subjected to experimental treatments. Lactate levels were quantified in 5 × 10⁵ cells using a lactate assay kit (BC2235, Solarbio) following the manufacturer’s instructions. Absorbance measurements were taken at 570 nm using a microplate reader.

### Glucose uptake assay

Cells (5 × 10⁴/well) were seeded in 6-well plates and glucose-starved for 1 h in serum-free medium. Cells were then incubated with 100 µM 2-[N-(7-nitrobenz-2-oxa-1,3-diazol-4-yl)amino]-2-deoxy-D-glucose (2-NBDG; MX4511, Jiqi) for 30 min at 37 °C. After washing with PBS, cells were fixed with 4% paraformaldehyde, mounted using antifade medium with DAPI (Vector Laboratories), and imaged using a fluorescence microscope. Fluorescence intensity was quantified using ImageJ software.

### Cell cycle analysis by flow cytometry

Cells were fixed in ice-cold 75% ethanol overnight at 4 °C. Following fixation, cells were washed twice with PBS and processed using a Cell Cycle Detection Kit (CA1510, Solarbio). Briefly, cells were resuspended in 500 µL PBS containing RNase A (50 µg/mL) and incubated at 37 °C for 30 min. Propidium iodide (PI; 50 µg/mL) was then added, and samples were incubated for 15 min at room temperature in the dark. Flow cytometry was performed on a BD FACSCanto II system, collecting ≥ 10,000 events per sample. Cell cycle distribution (G0/G1, S, and G2/M phases) was analyzed using FlowJo software.

### Statistical analysis

All statistical analyses were performed using GraphPad Prism software (version 9.5). Data were expressed as mean ± standard deviation (SD), with all in vitro experiments independently repeated at least 3 times and in vivo experiments conducted with *n* = 7 mice per group. Intergroup comparisons were conducted using unpaired Student’s t-tests for two-group analyses, while one-way or two-way analysis of variance (ANOVA) was employed for multi-group comparisons, as appropriate. *p* < 0.05 was considered statistically significant.

## Results

### Subsection OXA activated TLR4 signaling in ESCC cells

TLR4 is overexpressed in esophageal squamous cell carcinoma (ESCC) and correlates with poor patient survival [37]. While oxaliplatin (OXA) has been shown to upregulate TLR4 expression in dorsal root ganglion neurons, its role in mediating chemotherapeutic effects in ESCC remains unclear.

**Fig. 1:**
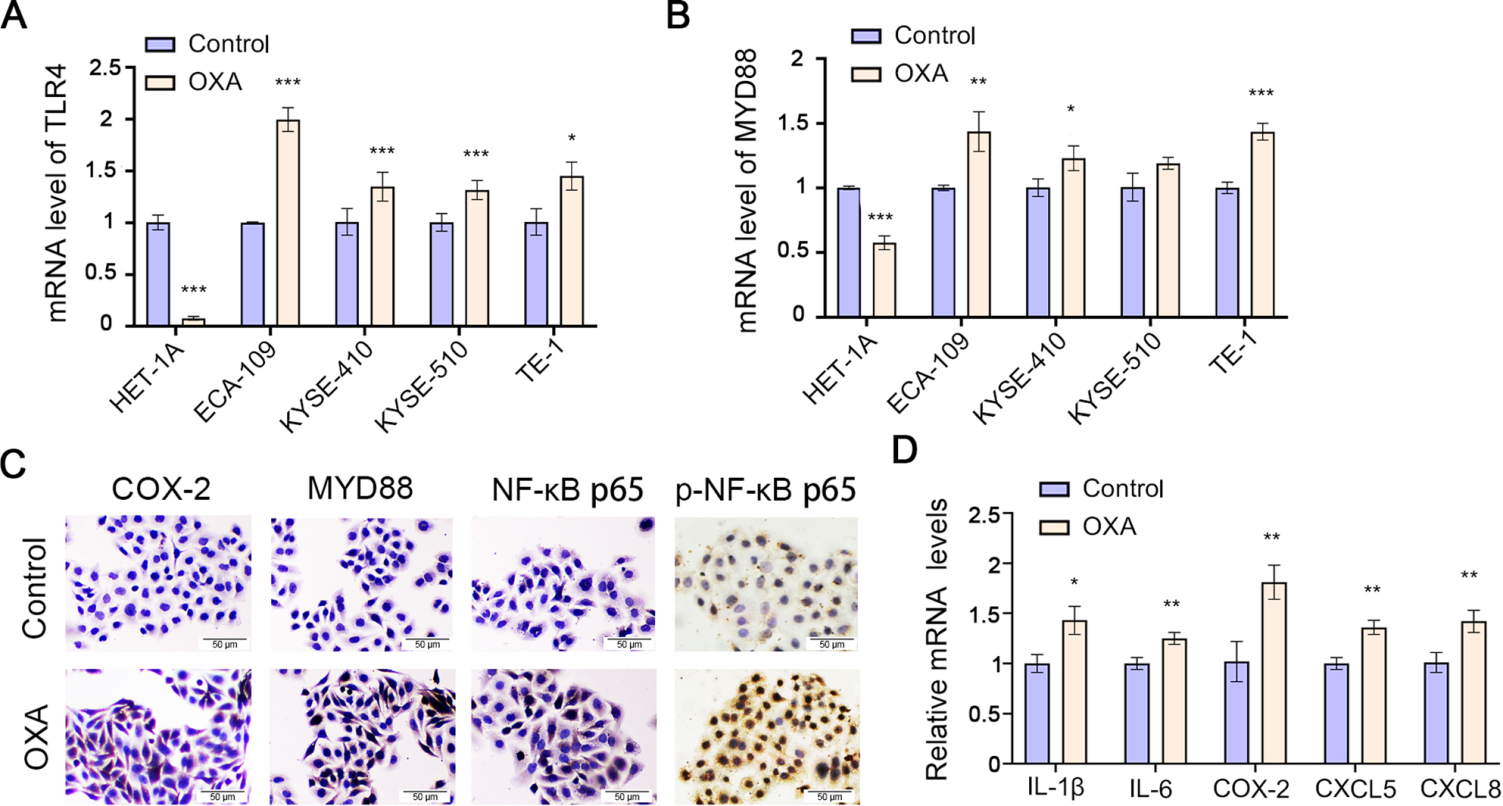
OXA activated TLR4 signaling in ESCC cells. The cells were treated by OXA (25 µM) for 24 h. **A**, **B**. The mRNA expressions of TLR4 and MYD88 in ESCC cell lines and normal esophageal cells were detected by qRT-PCR. **C**. ICC showed the immunocytochemical activity of NF-κB p65, p-NF-κB p65, COX-2, and MYD88. Scale bar: 50 μm. **D**. qRT-PCR analysis of mRNA levels of IL-1β, IL-6, COX-2, CXCL5, and CXCL8. *p < 0.05, **p < 0.01, ***p < 0.001 vs. Control group

Our investigation demonstrated that OXA treatment significantly elevated TLR4 mRNA levels in ESCC cell lines (ECA-109, KYSE-410, KYSE-510, and TE-1) compared to normal esophageal epithelial cells (HET-1A) (Fig. 1A). Furthermore, OXA upregulated MYD88 (a key TLR4 downstream adaptor) expression in ECA-109, KYSE-410, and TE-1 cells (Fig. 1B). Bioinformatic analysis of public datasets demonstrated elevated TLR4 expression in cisplatin-resistant ESCC (GSE229974) and OXA-resistant hepatocellular carcinoma cells (GSE206501) (Fig. S3). Given the most pronounced TLR4 induction in ECA-109 cells, we selected this line for subsequent experiments. Immunocytochemistry confirmed OXA-mediated upregulation of phosphorylated NF-κB p65, MYD88, and COX-2 (Fig. 1C), indicating TLR4-dependent activation of pro-inflammatory signaling. qRT-PCR analysis further revealed OXA-induced expression of inflammatory mediators (IL-1β, IL-6, COX-2) and chemokines (CXCL5, CXCL8) (Fig. 1D). Notably, CK14 overexpression is associated with poor prognosis (Chu et al. 2001), suggesting TLR4’s potential role in maintaining aggressive tumor phenotypes. Together, these findings suggest that OXA-induced TLR4 activation promotes tumor aggressiveness through NF-κB-mediated inflammatory signaling.

### TLR4 signaling inhibition potentiates OXA’s anti-tumor effects in ESCC cells

**Fig. 2:**
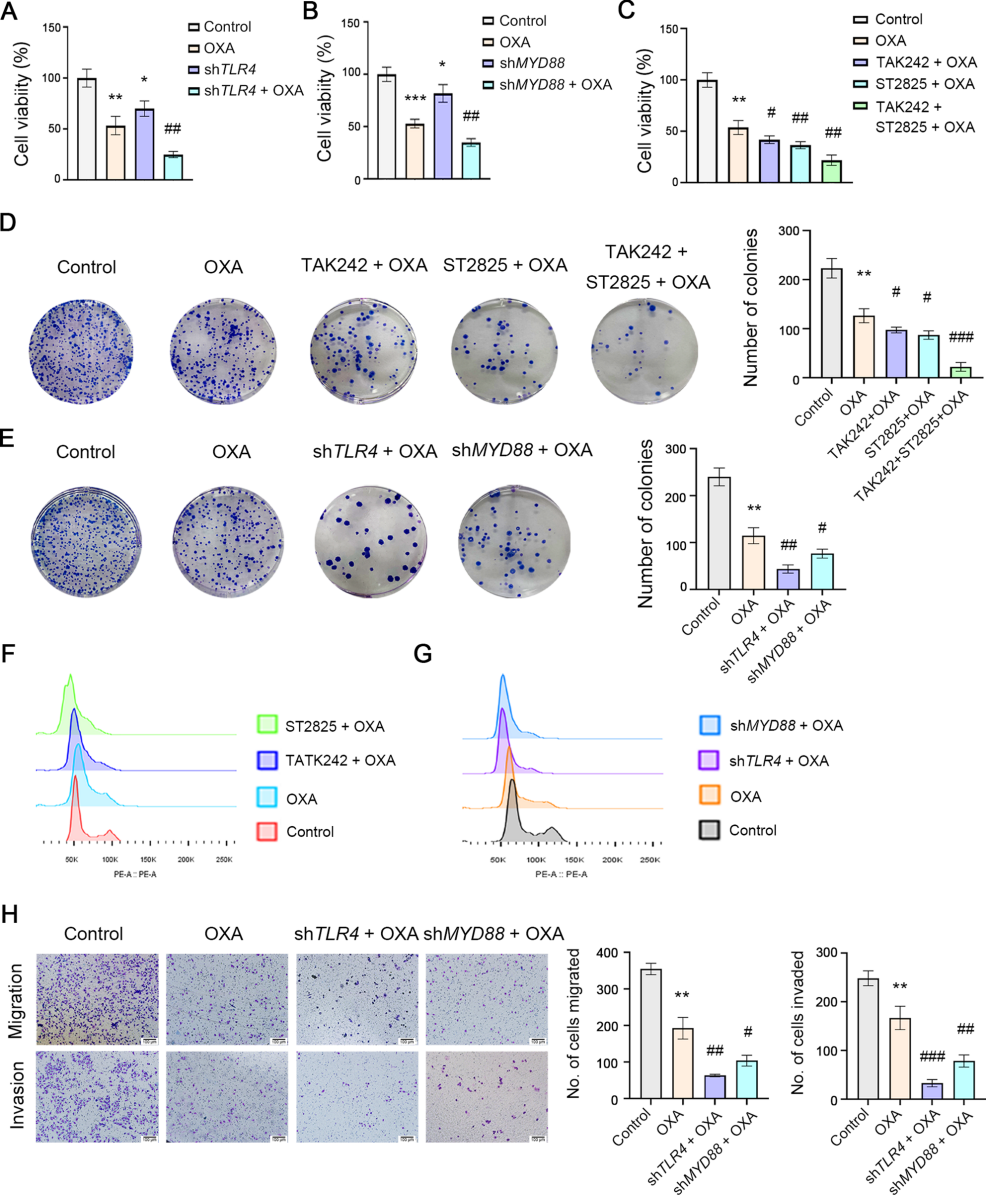
Inhibition of TLR4 signaling enhances the inhibitory effect of OXA on proliferation, migration, and invasion of ESCC cells. ECA-109 cells were treated by OXA (25 µM) for 24 h. **A-C**. Inhibition of TLR4 signal can enhance the inhibitory effect of OXA on cell viability. MTT assay was used to test cell viability. **D**,** E**. Colony formation assay was used to evaluate the proliferative capacity of ESCC cells. **F**, **G**. Flow cytometry was used to analyze the cell cycle of ESCC cells. **H**. Transwell migration and invasion assays were used to assess the migration and invasion abilities of ESCC cells. Scale bar: 100 μm. The concentration of TAK-242 is 100 nM. The concentration of ST2825 is 3 µM. ^*^p < 0.05, ^**^p < 0.01, ^***^p < 0.001, vs. Control group; ^#^*p* < 0.05, ^##^*p* < 0.01, ^###^*p* < 0.001, vs. OXA group

To investigate the functional role of TLR4 signaling in OXA-treated ESCC cells, we performed comprehensive analyses of cell proliferation, migration and invasion. Our MTT assays demonstrated that both OXA treatment and TLR4 silencing significantly reduced cell viability, with combined treatment showing synergistic inhibitory effects (Fig. 2A). Similar enhancement of OXA’s cytotoxic effects was observed upon MYD88 knockdown (Fig. 2B), and was recapitulated using pharmacological inhibitors (TAK-242 for TLR4; ST2825 for MYD88) (Fig. 2C).

Colony formation assays revealed that pharmacological inhibition of TLR4/MYD88 signaling significantly suppressed ESCC cell proliferation. Notably, combining TLR4 pathway inhibition with OXA treatment resulted in greater suppression of colony formation than either treatment alone (Fig. 2D). These findings were validated through genetic approaches, as TLR4 or MYD88 knockdown similarly enhanced OXA’s anti-proliferative effects (Fig. 2E). Cell cycle analysis further demonstrated that TLR4 inhibition promoted G0/G1 phase arrest, particularly when combined with OXA treatment (Fig. 2F-G).

In migration and invasion assays, genetic ablation of either TLR4 or MYD88 not only significantly impaired ESCC cell motility but also potentiated OXA’s inhibitory effects (Fig. 2H). These results collectively suggest that TLR4/MYD88 signaling blockade enhances OXA’s therapeutic efficacy across multiple oncogenic processes in ESCC.

### TLR4 signaling inhibition attenuates OXA-induced inflammatory response

**Fig. 3:**
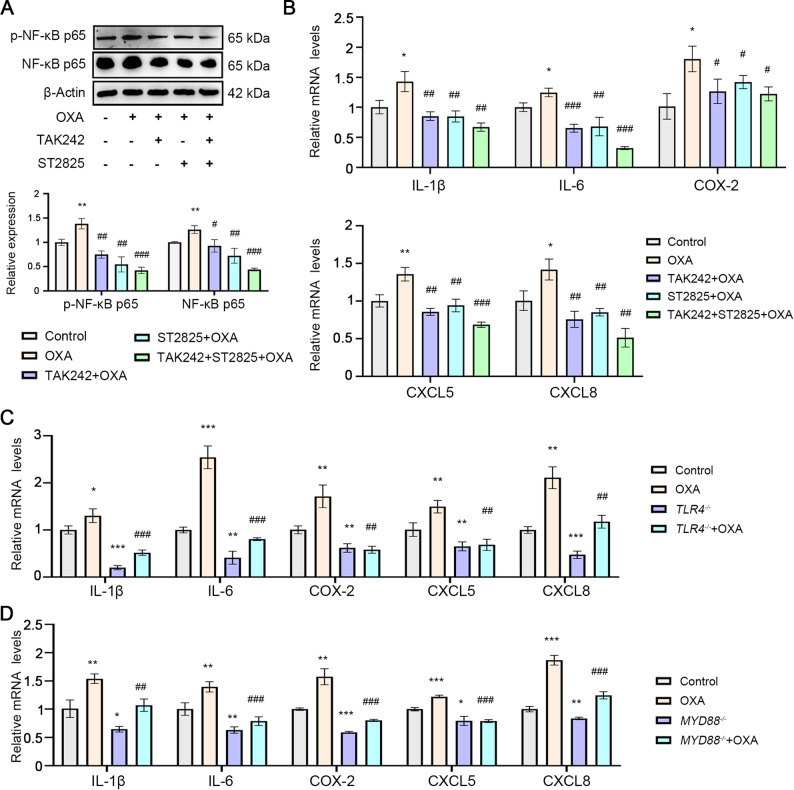
Inhibition of TLR4 signaling attenuates the inflammatory factors activated by OXA. ECA-109 cells were treated by OXA (25 µM) for 24 h. **A**. Western blot analysis revealed the protein levels of NF-κB p65 and phosphorylated NF-κB p65 in ESCC cells. **B**. qRT-PCR was used to detect the levels of inflammatory factors in ESCC cells. **C**, **D**. qRT-PCR was used to detect the mRNA levels of inflammatory factors in ESCC cells with knocked-down TLR4 or MYD88. The concentration of TAK-242 is 100 nM. The concentration of ST2825 is 3 µM. ^*^p < 0.05, ^**^p < 0.01, ^***^p < 0.001 vs. Control group; ^#^*p* < 0.05, ^##^*p* < 0.01, ^###^*p* < 0.001 vs. OXA group

 The TLR4/MYD88/NF-κB axis plays a pivotal role in mediating inflammatory responses and carcinogenesis. We first confirmed that OXA treatment activates NF-κB p65 phosphorylation (Fig. 3A), suggesting its potential to stimulate pro-tumorigenic inflammatory pathways during chemotherapy. Importantly, pharmacological inhibition of TLR4 (TAK-242) or MYD88 (ST2825) significantly attenuated OXA-induced NF-κB p65 activation (Fig. 3A). Consistent with NF-κB activation, OXA treatment markedly upregulated mRNA expression of pro-inflammatory cytokines (IL-1β, IL-6), inflammatory mediators (COX-2) and chemokines (CXCL5, CXCL8). Notably, TLR4 pathway inhibition reversed these OXA-induced effects (Fig. 3B). Genetic approaches further validated these findings, as both TLR4 and MYD88 knockdown effectively suppressed the inflammatory response triggered by OXA treatment (Fig. 3C-D). These results suggest that TLR4 signaling blockade can mitigate the chemotherapy-induced inflammatory microenvironment that may otherwise compromise treatment efficacy.

### TLR4 signaling inhibition attenuates OXA-induced glycolytic activity

**Fig. 4:**
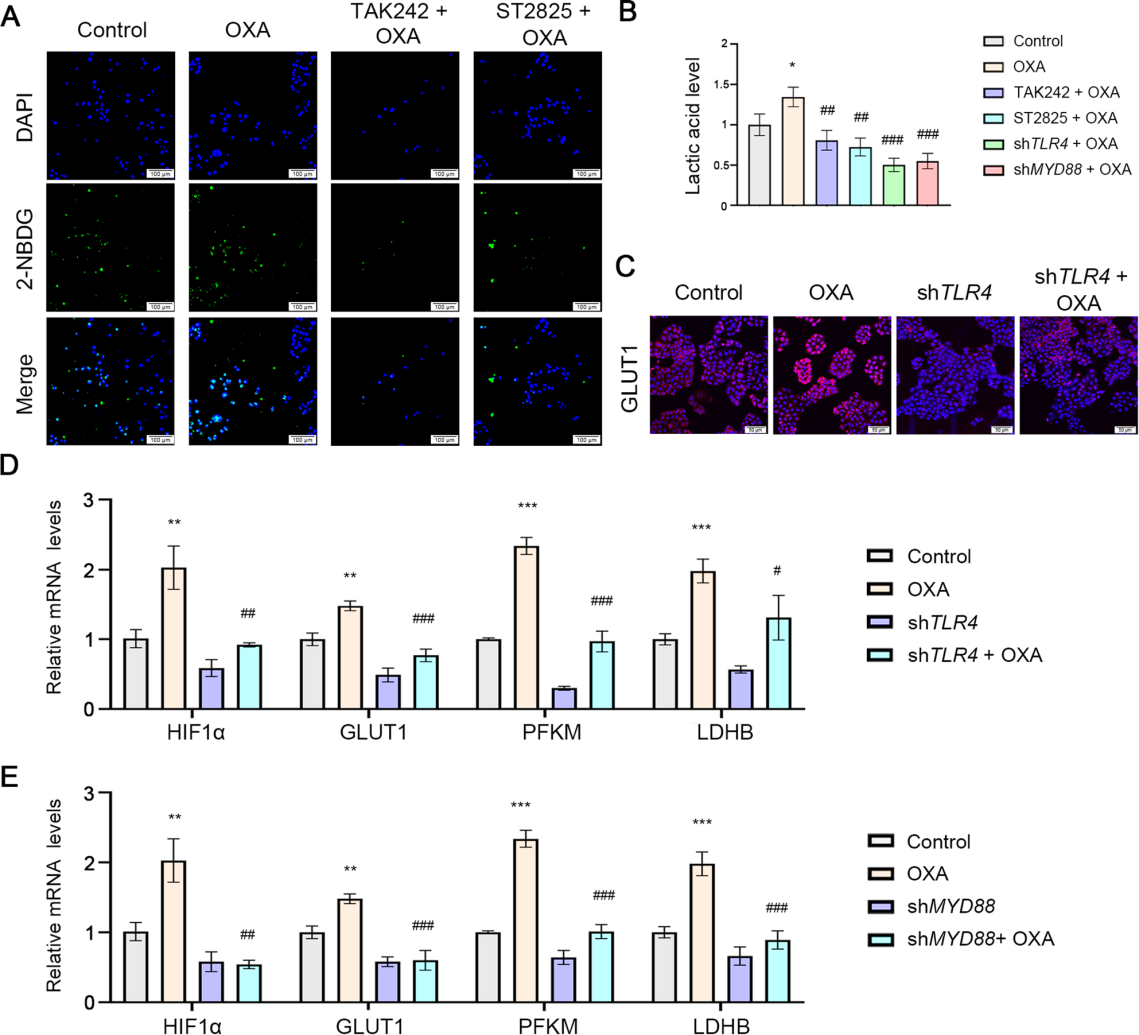
Inhibition of TLR4 signaling reduces glycolysis induced by OXA. ECA-109 cells were treated by OXA (25 µM) for 24 h. **A**. 2-NBDG assay was used to detect glucose uptake. Scale bar: 100 μm. **B**. Lactate levels were measured with a lactate assay kit. **C**. The expression of GLUT1 was detected by immunofluorescence. Scale bar: 50 μm. **D**, **E**. qRT-PCR was performed to detect the mRNA levels of HIF-1α, GLUT1, PFKM and LDHB in ESCC cells with knocked-down *TLR4* or *MYD88.* TAK-242 100 nM, ST2825 3 µM, OXA 25 µM. ^*^ *p* < 0.05, ^**^ *p* < 0.01, ^***^ *p* < 0.001 vs. Control group; ^#^*p* < 0.05, ^##^*p* < 0.01, ^###^*p* < 0.001 vs. OXA group

Previous studies have demonstrated that TLR4 enhances glycolysis through the HIF-1α/GLUT1 axis, thereby reducing therapeutic sensitivity [29]. In the current study, pharmacological inhibition of TLR4 signaling using TAK-242 (a TLR4-specific inhibitor) and ST2825 (a MYD88 inhibitor) significantly attenuated OXA-induced glycolytic activity. Specifically, both inhibitors markedly reduced intracellular glucose uptake as measured by 2-NBDG labeling (Fig. 4A) and decreased lactate production (Fig. 4B). These findings were further corroborated through genetic approaches, where siRNA-mediated knockdown of either TLR4 or MYD88 similarly suppressed lactate generation (Fig. 4B). Immunofluorescence analysis revealed that TLR4 knockdown substantially diminished GLUT1 expression (Fig. 4C), while qPCR analysis demonstrated downregulation of key glycolytic regulators including HIF-1α, GLUT1, PFKM and LDHB at the transcriptional level following TLR4 or MYD88 silencing (Fig. 4D-E). Collectively, these results strongly suggest that TLR4 signaling modulates OXA chemosensitivity through regulation of glycolytic metabolism in ESCC cells.

### TLR4 knockout enhances oxaliplatin chemosensitivity in ESCC in vivo

**Fig. 5:**
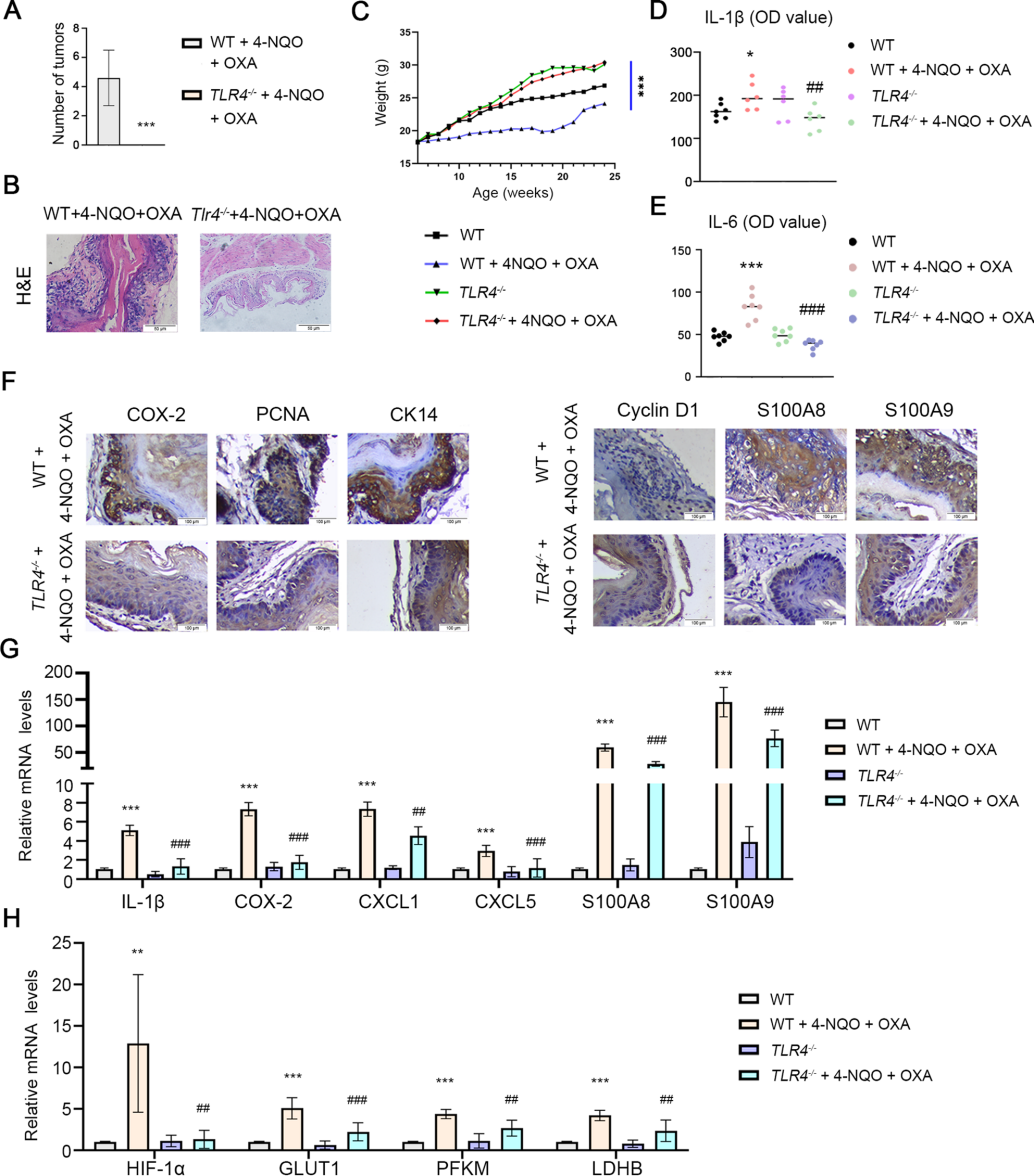
TLR4 knockout enhances the sensitivity of OXA chemotherapy in ESCC in vivo. **A**. Number of tumors. **B**. HE staining of esophageal epithelial tissues. Scale bar: 50 μm. **C**. Body weight of mice. **D**, **E**. the levels of serum IL-1β and IL-6 in mice from different groups were detected by ELISA. **F**. The immunohistochemical activities of PCNA, CK14, Cyclin D1, COX-2, S100A8 and S100A9 in the esophageal tissue were detected. Scale bar: 100 μm. **G**, **H**. The mRNA levels of inflammatory cytokines and glycolysis-related proteins in esophageal tissue were detected by qRT-PCR. ^*^p < 0.05, ^**^p < 0.01, ^***^p < 0.001 vs. WT group; ^#^*p* < 0.05, ^##^*p* < 0.01, ^###^*p* < 0.001 vs. WT + 4NQO + OXA group

To validate our in vitro observations, we established a 4-NQO-induced ESCC mouse model. Compared to wild-type (WT) controls, TLR4 knockout (TLR4-/-) mice showed significantly attenuated oxaliplatin (OXA)-induced weight loss (Fig. 5C), suggesting reduced treatment-related systemic toxicity. Histopathological examination revealed that OXA treatment more effectively ameliorated esophageal hyperplasia in TLR4-/- mice (Fig. 5B). The genetic ablation of TLR4 significantly enhanced OXA’s antitumor efficacy, as evidenced by reduced tumor formation without significant loss of body weight in mice (Fig. 5A and C) and decreased serum levels of OXA-induced proinflammatory cytokines IL-1β and IL-6 (Fig. 5D and E). Immunohistochemical analysis demonstrated that TLR4 knockout suppressed OXA-induced expression of cellular proliferation markers (PCNA and CK14), endogenous TLR4 ligands (S100A8 and S100A9), and key cell cycle regulators (COX-2 and Cyclin D1) (Fig. 5F). qRT-PCR analysis further confirmed significant downregulation of inflammatory mediators (IL-6, COX-2), metabolic regulators (HIF-1α, GLUT1, PFKM, LDHB) and Chemotactic factors (S100A8, S100A9, CXCL1, CXCL5) (Fig. 5G and H). These results collectively support that TLR4 knockout enhances oxaliplatin chemosensitivity in ESCC through coordinated modulation of inflammatory responses and glycolytic metabolism.

## Discussion

Oxaliplatin (OXA) has emerged as a promising antineoplastic agent for esophageal cancer treatment [38–40], however, its clinical efficacy remains limited by the development of chemoresistance. Our study identifies TLR4 as a critical regulator of OXA sensitivity, coordinating a dual mechanism involving both inflammatory responses and metabolic reprogramming that promotes tumor survival. Through comprehensive in vitro and in vivo investigations, we show that pharmacological or genetic inhibition of TLR4 signaling potentiates OXA’s antitumor effects, providing a novel combinatorial therapeutic approach for esophageal squamous cell carcinoma.

Our study demonstrates that TLR4 is selectively upregulated in OXA-treated ESCC cells but not in normal esophageal epithelial cells, revealing a tumor-specific pathway activation pattern. NF-κB, the central downstream effector of TLR4/MYD88 signaling and a master regulator of inflammatory and differentiation-related genes [41], mediates OXA-induced upregulation of pro-inflammatory cytokines (IL-1β, IL-6, COX-2), chemokines (CXCL5, CXCL8), and the poor prognostic marker CK14 [42], collectively establishing an immunosuppressive tumor microenvironment. Beyond the intrinsic inflammatory responses of tumor cells, the inflammatory landscape of the tumor microenvironment (TME)—particularly tumor-associated macrophages (TAMs) and tumor-associated neutrophils (TANs)—serves as a critical mediator of chemoresistance [43]. Importantly, our study demonstrates that TLR4/NF-κB signaling specifically upregulates the chemokine CXCL8, a well-documented chemoattractant for both TAMs and TANs [44, 45]. These results suggest that tumor cell–intrinsic TLR4 activation functions as an inflammatory signaling hub, facilitating immune cell recruitment and polarization within the TME, thereby reinforcing chemotherapy resistance.

Importantly, pharmacological or genetic inhibition of TLR4/MYD88 signaling effectively suppressed NF-κB p65 phosphorylation, downregulated proliferative markers (PCNA, cyclin D1), and enhanced OXA-induced G0/G1 phase arrest, demonstrating that TLR4 blockade disrupts both cell cycle progression and metastatic potential. These in vitro findings were further validated in vivo, where TLR4 knockout not only attenuated OXA-induced systemic toxicity but also significantly improved therapeutic response by reducing esophageal hyperplasia, underscoring the clinical relevance of targeting TLR4 signaling to enhance OXA chemosensitivity in ESCC.

Emerging evidence indicates that TLR4 orchestrates crucial metabolic adaptations beyond its inflammatory functions, creating a multifaceted mechanism of chemoresistance. Cancer cells undergo glucose metabolic reprogramming to meet heightened bioenergetic and biosynthetic demands while mitigating oxidative stress, processes essential for tumor proliferation and survival [46, 47]. This metabolic shift mirrors observations in LPS-activated macrophages, where TLR4 signaling modulates glucose metabolism through HIF-1α and IL-1β regulation [48, 49]. Our study extends these findings to ESCC, demonstrating that OXA treatment activates a TLR4/HIF-1α axis that drives glycolytic flux, a phenomenon previously documented in hepatocellular carcinoma. Notably, TLR4 inhibition reversed OXA-induced metabolic changes by reducing glucose uptake and lactate production, downregulating key glycolytic enzymes (GLUT1, PFKM, LDHB), attenuating HIF-1α expression. These enzymes collectively sustain the Warburg effect, enabling tumor cells to circumvent OXA-mediated metabolic stress.

**Fig. 6:**
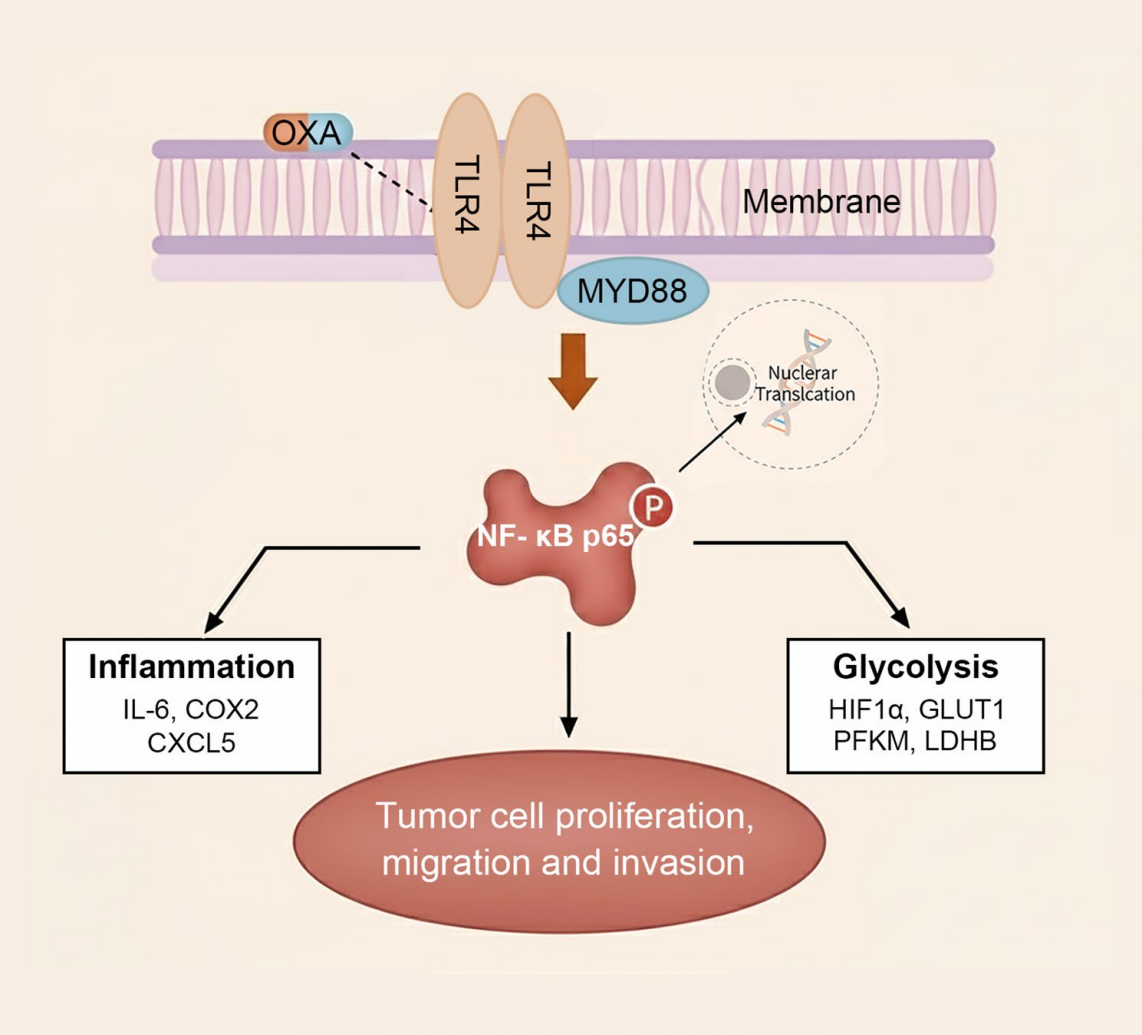
Schematic diagram illustrating the mechanism by which TLR4 inhibition enhances oxaliplatin (OXA) chemosensitivity in esophageal squamous cell carcinoma (ESCC). OXA treatment upregulates TLR4 and its downstream adaptor protein MYD88, which activates the phosphorylation of NF-κB p65. This activation drives two parallel pathways: (1) the inflammatory response, characterized by the upregulation of pro-inflammatory factors such as IL-6, COX-2, and CXCL5; (2) the glycolytic metabolic reprogramming, mediated by the HIF-1α/GLUT1 axis and enhanced expression of glycolytic enzymes including PFKM and LDHB. These two pathways synergistically promote ESCC cell proliferation, migration, and invasion, ultimately reducing OXA chemosensitivity. Inhibition of TLR4 (via genetic knockout, shRNA knockdown, or pharmacological inhibitor TAK-242) or its downstream mediator MYD88 (via shRNA knockdown or inhibitor ST2825) blocks NF-κB p65 phosphorylation, thereby suppressing both the inflammatory response and glycolytic activity. This dual inhibition disrupts the adaptive survival mechanisms of ESCC cells, potentiating the anti-tumor efficacy of OXA

Particularly intriguing is the potential crosstalk between TLR4-driven metabolic and inflammatory pathways. HIF-1α and GLUT1 play central roles in regulating glucose metabolism, and inhibition of HIF-1α has been shown to disrupt the pentose phosphate pathway, reduce NADPH generation, and weaken cellular antioxidant capacity, thereby sensitizing tumor cells to oxidative stress [50]. In this context, Zhou et al. developed a tumor microenvironment–responsive nanomedicine capable of amplifying reactive oxygen species (ROS) for photodynamic immunotherapy [51]. A potential synergistic mechanism may therefore exist between metabolic targeting strategies and ROS-amplifying therapies, whereby suppression of glycolysis compromises NADPH-dependent ROS scavenging and lowers the threshold for ROS-induced tumor cell death. Moreover, lactate accumulation, a consequence of enhanced glycolysis, may stabilize HIF-1α and subsequently amplify NF-κB-mediated cytokine production [52], may create a self-reinforcing loop that perpetuates tumor survival under chemotherapy pressure (Fig. 6).

Our in vivo findings demonstrate compelling evidence for TLR4 targeting as a strategy to enhance OXA efficacy in ESCC treatment. TLR4 knockout mice showed three key therapeutic benefits: (1) significant reduction in tumor burden, (2) attenuation of pro-tumorigenic inflammation, and (3) normalization of glucose metabolic pathways, all consistent with our in vitro mechanistic studies. These results corroborate emerging clinical evidence that TLR4 pathway inhibition can potentiate chemotherapy response across various malignancies [53, 54].

Despite the therapeutic potential of the TLR4/NF-κB/HIF-1α axis in ESCC, clinical translation faces two primary hurdles. First, the pharmacological selection of specific TLR4 inhibitors remains challenging. While TAK-242 effectively enhances oxaliplatin sensitivity in preclinical models, its clinical utility is often limited by poor bioavailability and potential off-target effects [55]. Second, systemic TLR4 inhibition carries the risk of compromising the innate immune system. potentially leading to impaired anti-infective defenses or immune paralysis [56]. To mitigate these risks, future strategies should prioritize localized delivery system**s**, such as tumor-targeted nanoparticles, to concentrate the inhibitory effects within the tumor microenvironment while minimizing systemic toxicity.

Several limitations of this study should also be acknowledged. Although our findings were validated using cell lines and genetically engineered mouse models, patient-derived samples and clinical datasets were not included. In addition, the complex interactions between tumor cells and immune populations in the human tumor microenvironment cannot be fully recapitulated in the current models. Future studies incorporating clinical specimens and immune-competent systems will be necessary to further validate these findings.

## Conclusions

Our study identifies TLR4 as a key mediator linking inflammation and metabolism in OXA-treated ESCC. Inhibition of TLR4 signaling not only mitigates OXA-induced cytotoxicity but also disrupts the tumor’s adaptive survival mechanisms. These findings provide a preclinical rationale for combining TLR4 inhibitors with OXA-based therapies and suggest potential dual-targeting strategies to improve outcomes in ESCC patients.

## Supplementary Information

Supplementary Material 1.

Supplementary Material 2.

Supplementary Material 3.

Supplementary Material 4.

Supplementary Material 5.

## Data Availability

All raw data and analysis scripts are available from the corresponding author upon reasonable request. Publicly available datasets analyzed in this study can be accessed from the Gene Expression Omnibus (GEO) under the following accession numbers: GSE229974 and GSE206501.
