# Supplementary material for: Inhibition of TLR4 enhances oxaliplatin chemotherapy sensitivity in esophageal squamous cell carcinoma by suppressing inflammation and glycolysis
Source: BMC Gastroenterol. 2026 Feb 10;26:168. doi: 10.1186/s12876-026-04663-2 (PMC12990394; doi:10.1186/s12876-026-04663-2)
Supplement: Supplementary file 1 — Supplementary Material 1. [file 12876_2026_4663_MOESM1_ESM.docx]

**Inhibition of TLR4 Enhances Oxaliplatin Chemotherapy Sensitivity in Esophageal Squamous Cell Carcinoma by Suppressing Inflammation and Glycolysis**

Ziqi Zhu^1, 2^, Meng Zhang^1^, Zequn Di^1^, Xin Tao^1^, Yaohui Dai^1^, Zhiqiang Zhan^3*^, Hongping Chen^1,2*^

1 Department of Histology and Embryology, Jiangxi Medical College, Nanchang University, Nanchang, Jiangxi, China

2 The MOE Basic Research and Innovation Center for the Targeted Therapeutics of Solid Tumors, School of Basic Medicine, Nanchang University, Nanchang, Jiangxi, China Affiliation 2;

3 Pingxiang People's Hospital. No. 8 Wugong Mountain Avenue, Pingxiang City, Jiangxi Province；337000

* Correspondence: Hongping Chen, School of Histology and Embryology, Jiangxi Medical College, Nanchang University, Nanchang, Jiangxi, China. Email address: jxchp2000@126.com;

Zhiqiang Zhan, Department of Oncology, Pingxiang People's Hospital. No. 8 Wugong Mountain Avenue, Pingxiang City, Jiangxi Jiangxi China. Email: zhanzhiqiang1982@foxmail.com

**Supplementary information**

Supplementary Fig. 1 Determination of drug sensitivity and selection of working concentrations. A–C Dose-response curves and half-maximal inhibitory concentration (IC₅₀) values for oxaliplatin (OXA), TAK242, and ST2825 in human ESCC ECA-109 cells, as determined by MTT viability assays. Results are expressed as mean ± SD. n ≥ 3 in each group. * *p* < 0.05, ** *p* < 0.01, *** *p* < 0.001, vs Control group.

Supplementary Fig. 2 Efficiency of virus infection. A. TLR4 and MYD88 expression in Eca-109 cells transfected with shTLR4 and shMYD88 by qRT-PCR and B western blot. Results are expressed as mean ± SD. n ≥ 3 in each group. * *p* < 0.05, ** *p* < 0.01, *** *p* < 0.001, vs Control group.

Supplementary Fig. 3 TLR4 expression in in cisplatin- or oxaliplatin-resistant patients. A. TLR4 expression in cisplatin-resistant ESCC patients from the GSE229974 dataset. B. TLR4 expression in in oxaliplatin-resistant liver cancer patients from the GSE206501 dataset. Results are expressed as mean ± SD. n = 3 in each group. * *p* < 0.05, ** *p* < 0.01, *** *p* < 0.001, vs Control group.

**Supplementary Table 1**. Primer sequences used for quantitative qRT-PCR. H: human; M: mouse.

| **Gene** |  | **Primer sequences (5'-3')** |
| --- | --- | --- |
| **H-ACTB** | **F** | **CACCTTCTACAATGAGCTGCG** |
|  | **R** | **ATAGCACAGCCTGGATAGCAAC** |
| **H-HIF1a** | **F** | **GAACGTCGAAAAGAAAAGTCTCG** |
|  | **R** | **CCTTATCAAGATGCGAACTCACA** |
| **H-GLUT1** | **F** | **TCTGGCATCAACGCTGTCTTC** |
|  | **R** | **CGATACCGGAGCCAATGGT** |
| **H-PFKM** | **F** | **GGTGCCCGTGTCTTCTTTGT** |
|  | **R** | **AAGCATCATCGAAACGCTCTC** |
| **H-LDHB** | **F** | **TCTGTGACCGCCAATTCTAAGA** |
|  | **R** | **GCACCAGATTGAGCCGACTC** |
| **H-TLR4** | **F** | **TTGGACAGTTTCCCACATTGA** |
|  | **R** | **AAGCATTCCCACCTTTGTTGG** |
| **H-MYD88** | **F** | **ACAGGCACCAGCATACA** |
|  | **R** | **TGGGTCCTTTCCAGAGT** |
| **H-IL-1B** | **F** | **AGTGGCAATGAGGATGA** |
|  | **R** | **GTAGTGGTGGTCGGAGA** |
| **H-IL-6** | **F** | **GGAGACTTGCCTGGTGA** |
|  | **R** | **CATTTGTGGTTGGGTCA** |
| **H-COX2** | **F** | **TCCTATTATACTAGAGCCCTTCCT** |
|  | **R** | **TTCCACAATCTCATTTGAATCAGG** |
| **H-CXCR2** | **F** | **CCTGTCTTACTTTTCCGAAGGAC** |
|  | **R** | **TTGCTGTATTGTTGCCCATGT** |
| **H-CXCL5** | **F** | **AGCTGCGTTGCGTTTGTTTAC** |
|  | **R** | **TGGCGAACACTTGCAGATTAC** |
| **H-CXCL8** | **F** | **ACTGAGAGTGATTGAGAGTGGAC** |
|  | **R** | **AACCCTCTGCACCCAGTTTTC** |
| **H-CK14** | **F** | **GGAGATGATTGGCAGCGTGGAG** |
|  | **R** | **AGAACTGGGAGGAGGAGAGGTG** |
| **M-ACTB** | **F** | **GTGACGTTGACATCCGTAAAGA** |
|  | **R** | **GCCGGACTCATCGTACTCC** |
| **M-S100A8** | **F** | **AAATCACCATGCCCTCTACAAG** |
|  | **R** | **CCCACTTTTATCACCATCGCAA** |
| **M-S100A9** | **F** | **GCACAGTTGGCAACCTTTATG** |
|  | **R** | **TGATTGTCCTGGTTTGTGTCC** |
| **M-CXCL1** | **F** | **CTGGGATTCACCTCAAGAACATC** |
|  | **R** | **CAGGGTCAAGGCAAGCCTC** |
| **M-COX2** | **F** | **TTCCAATCCATGTCAAAACCGT** |
|  | **R** | **AGTCCGGGTACAGTCACACTT** |
| **M-CXCR2** | **F** | **ATGCCCTCTATTCTGCCAGAT** |
|  | **R** | **GGTGCTCCGGTTGTATAAGATGA** |
| **M-IL-6** | **F** | **TTGGTCCTTAGCCACTCCTTC** |
|  | **R** | **GAATTGCCATTGCACAACTCTTT** |
| **M-GLUT1** | **F** | **GCAGTTCGGCTATAACACTGG** |
|  | **R** | **GCGGTGGTTCCATGTTTGATTG** |
| **M-HIF1a** | **F** | **GATGACGGCGACATGGTTTAC** |
|  | **R** | **CTCACTGGGCCATTTCTGTGT** |
| **M-PFKM** | **F** | **GAGCGAGAAGGACGACTCC** |
|  | **R** | **GCCTCCGATGACACACAGA** |
| **M-LDHB** | **F** | **TGCGTCCGTTGCAGATGAT** |
|  | **R** | **TTTCGGAGTCTGGAGGAACAA** |
