# Supplementary figures and images for: Inhibition of TLR4 enhances oxaliplatin chemotherapy sensitivity in esophageal squamous cell carcinoma by suppressing inflammation and glycolysis
Source: BMC Gastroenterol. 2026 Feb 10;26:168. doi: 10.1186/s12876-026-04663-2 (PMC12990394; doi:10.1186/s12876-026-04663-2)

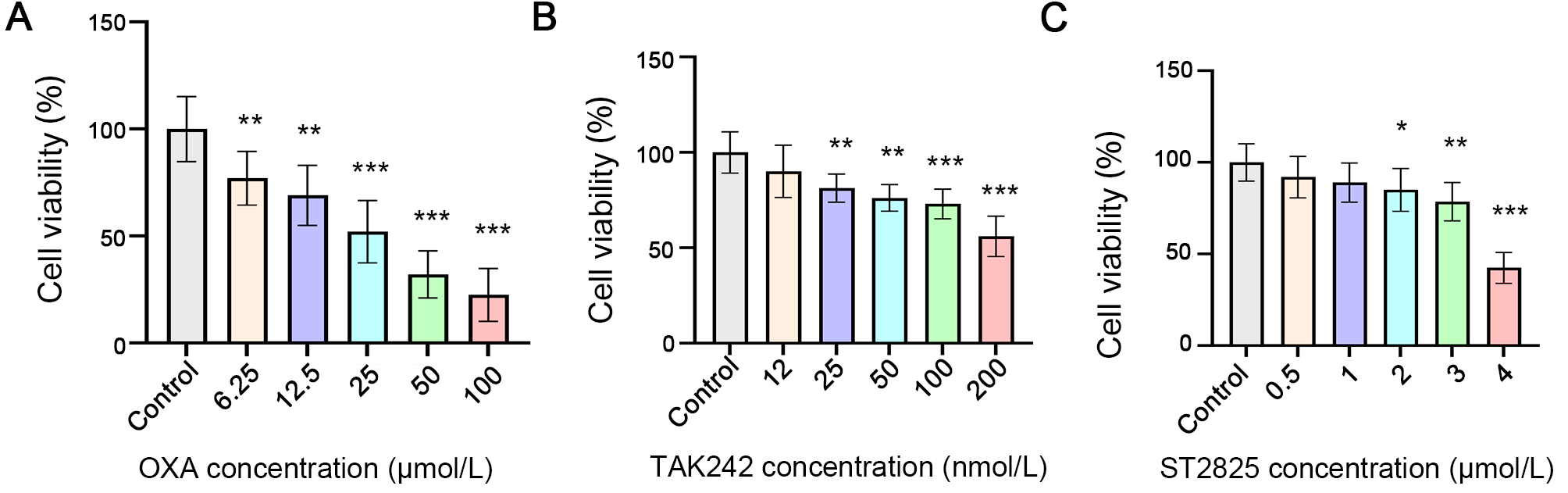

Supplement: Supplementary file 2 — Supplementary Material 2. [file 12876_2026_4663_MOESM2_ESM.tif]

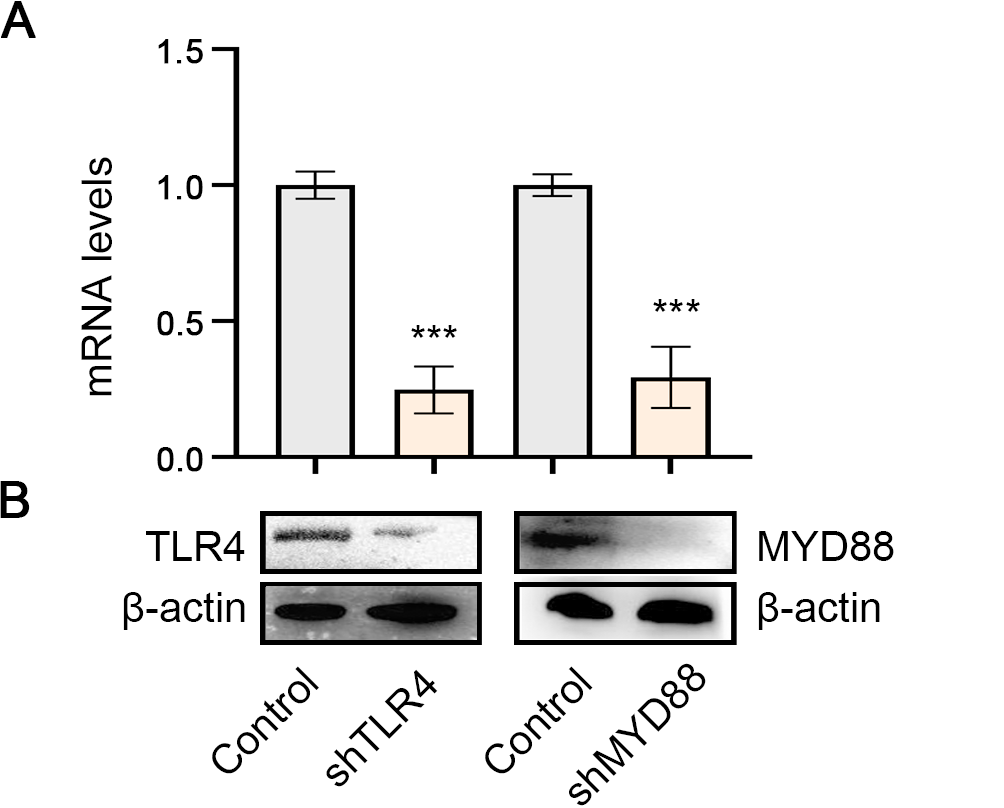

Supplement: Supplementary file 3 — Supplementary Material 3. [file 12876_2026_4663_MOESM3_ESM.tif]

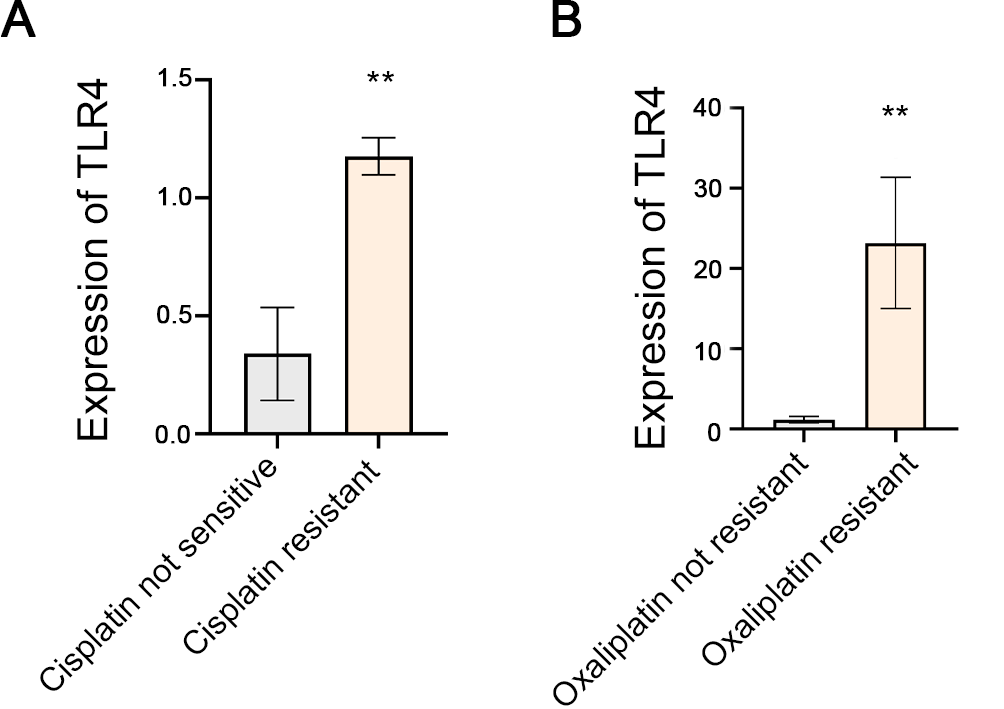

Supplement: Supplementary file 4 — Supplementary Material 4. [file 12876_2026_4663_MOESM4_ESM.tif]

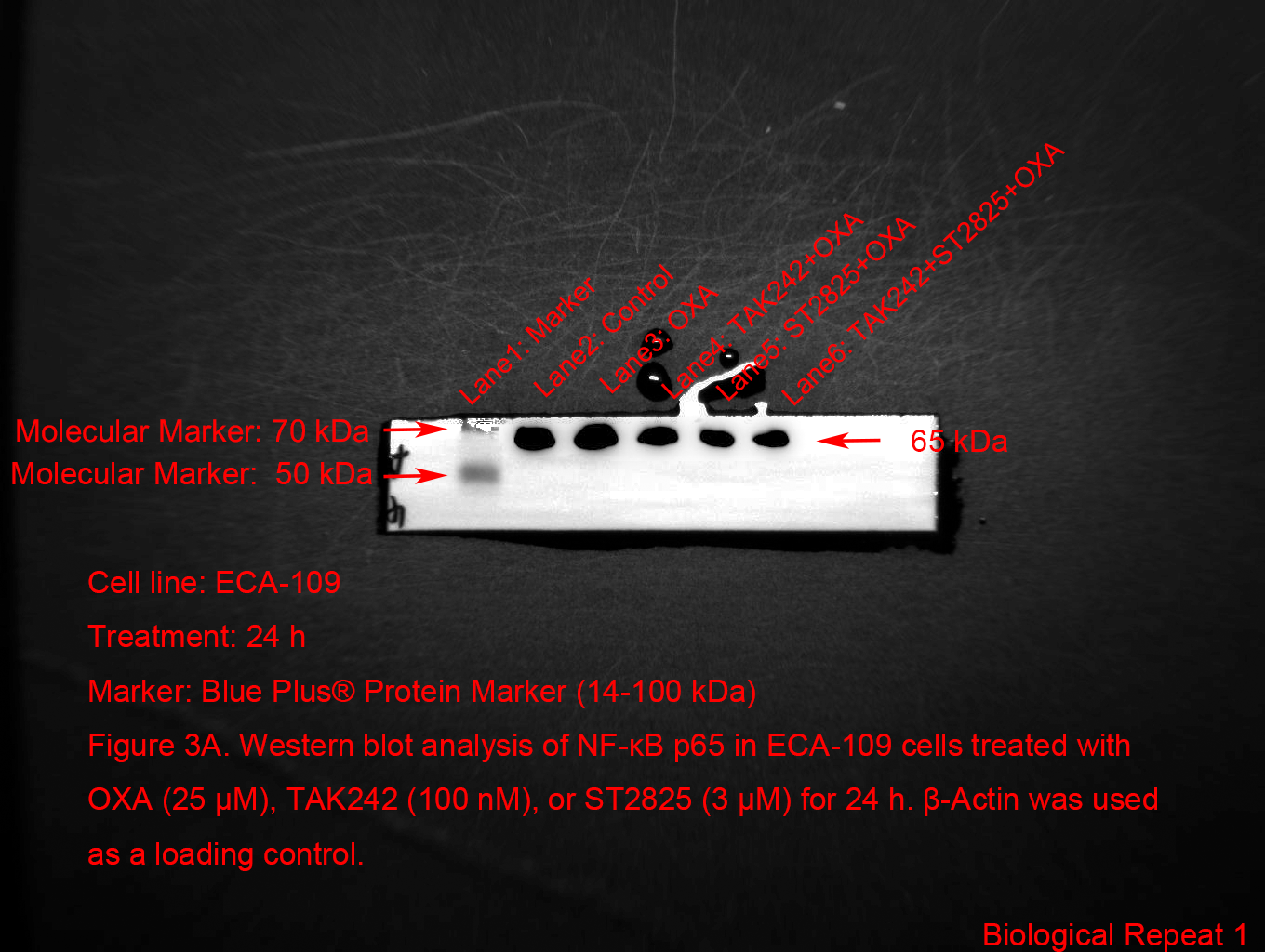

Supplement: Supplementary file 5 — Supplementary Material 5. [file 12876_2026_4663_MOESM5_ESM.zip › uncropped GEL/Suppl_Uncropped_WB_Fig3A _NF-κB p65_Repeat1.tif]

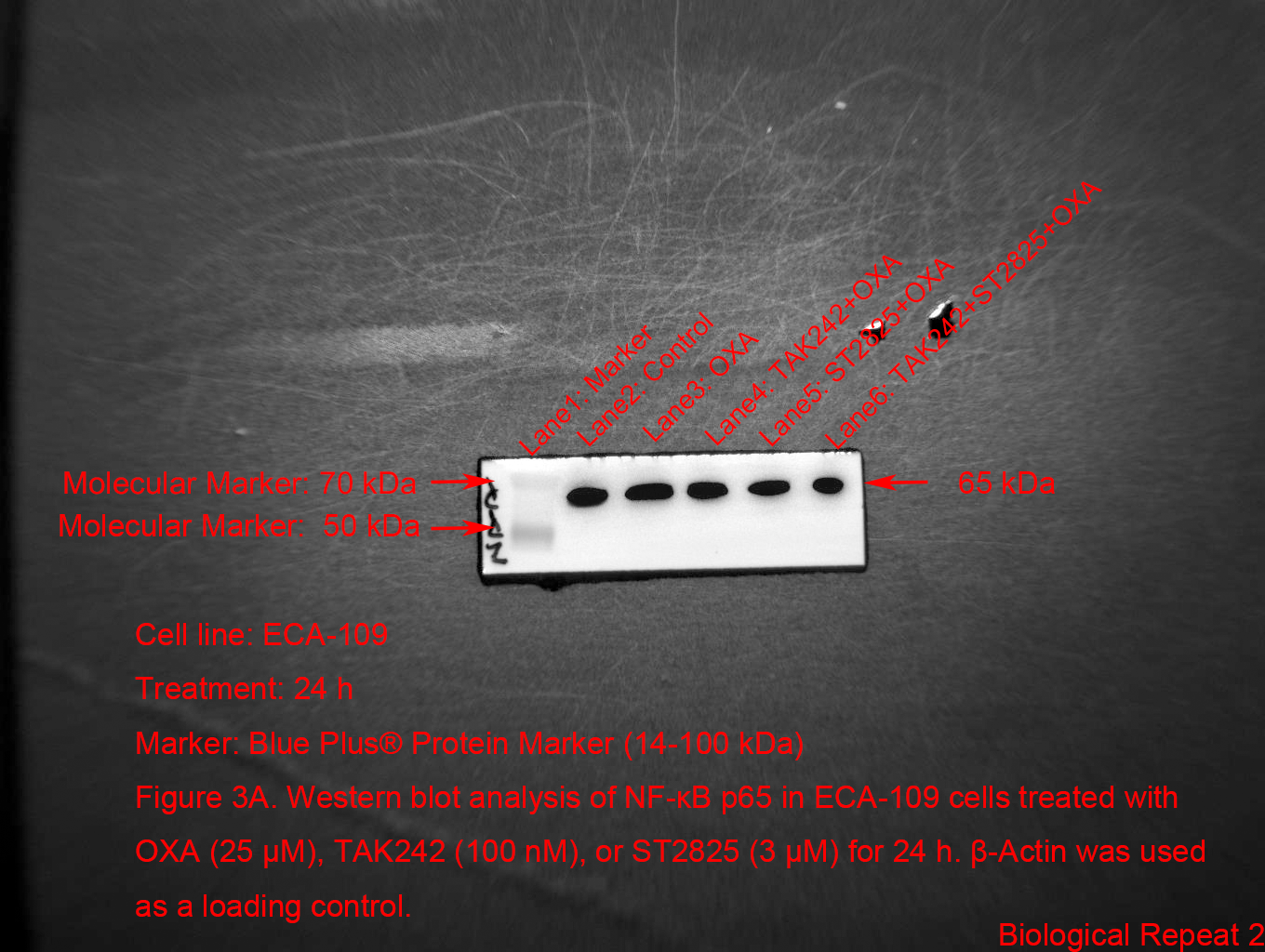

Supplement: Supplementary file 5 — Supplementary Material 5. [file 12876_2026_4663_MOESM5_ESM.zip › uncropped GEL/Suppl_Uncropped_WB_Fig3A _NF-κB p65_Repeat2.tif]

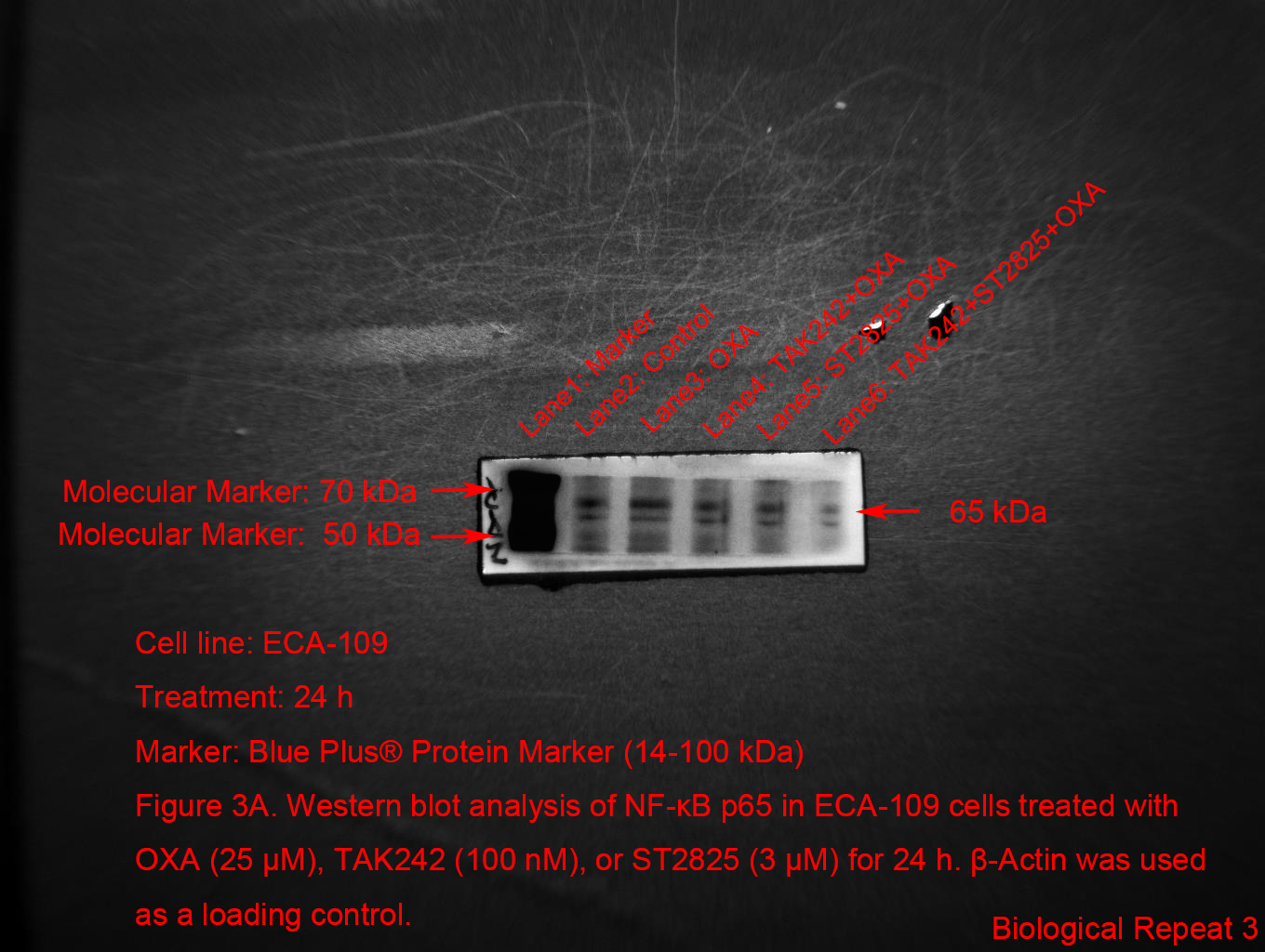

Supplement: Supplementary file 5 — Supplementary Material 5. [file 12876_2026_4663_MOESM5_ESM.zip › uncropped GEL/Suppl_Uncropped_WB_Fig3A _NF-κB p65_Repeat3.tif]

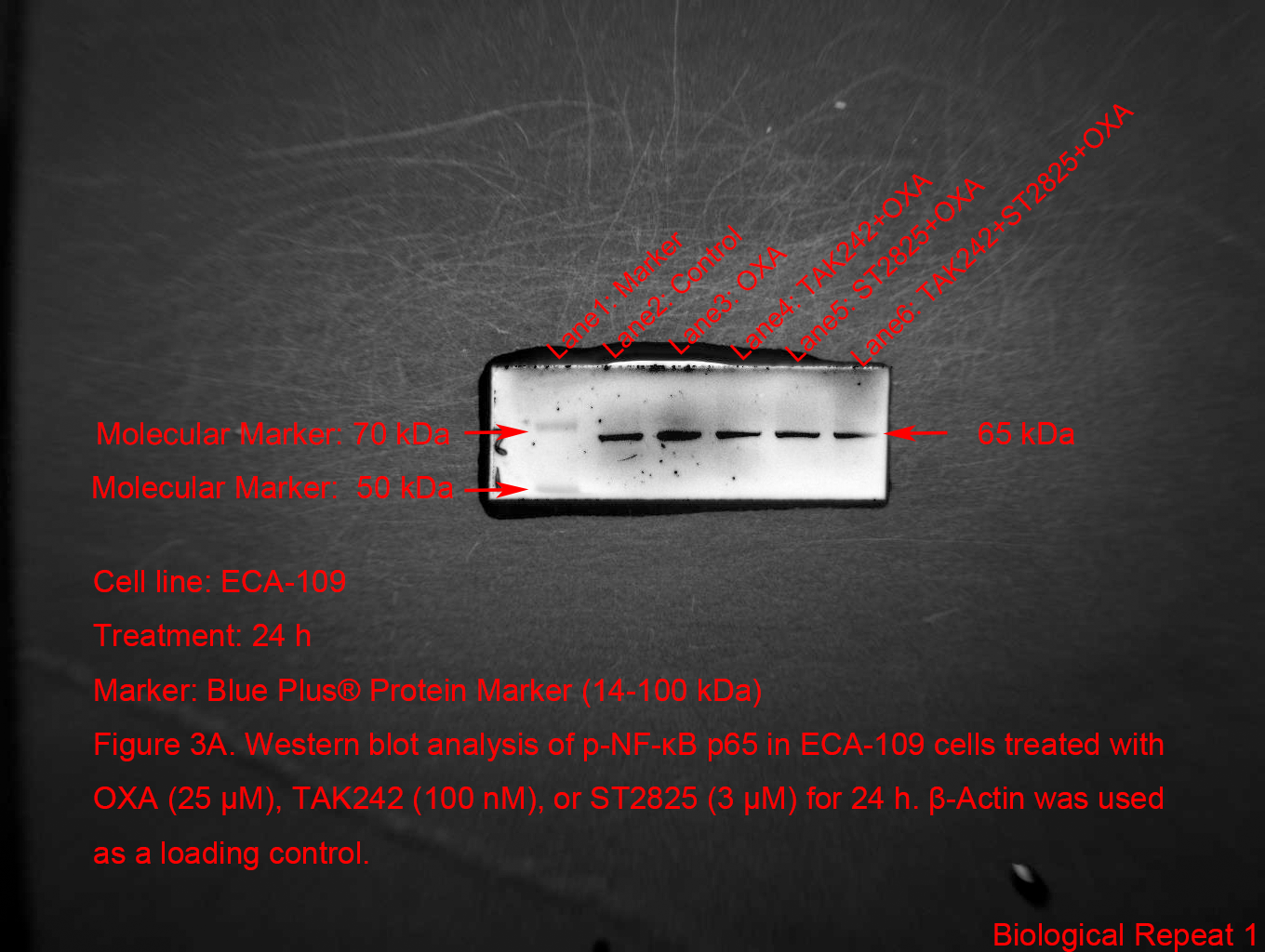

Supplement: Supplementary file 5 — Supplementary Material 5. [file 12876_2026_4663_MOESM5_ESM.zip › uncropped GEL/Suppl_Uncropped_WB_Fig3A _p-NF-κB p65_Repeat1.tif]

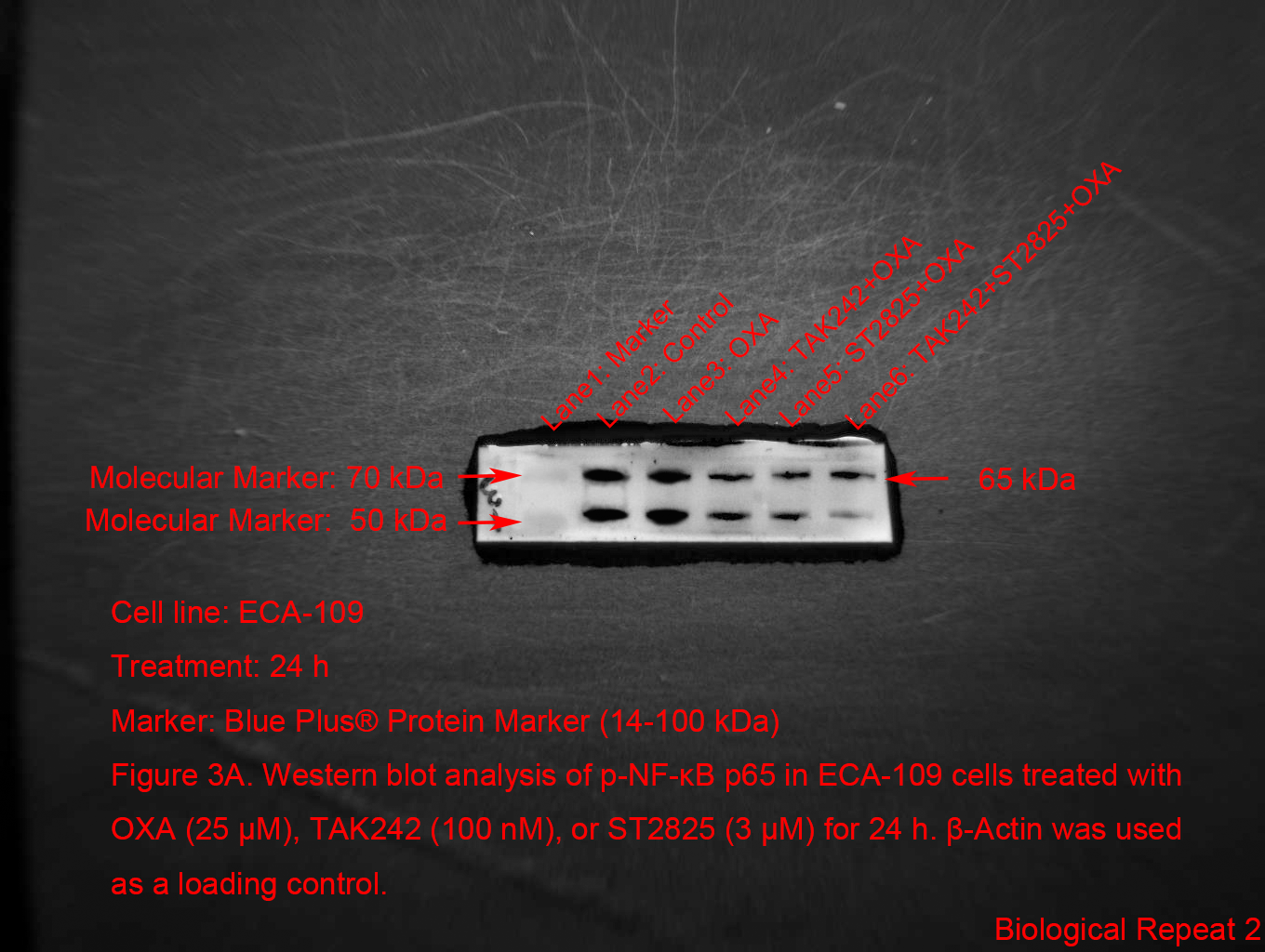

Supplement: Supplementary file 5 — Supplementary Material 5. [file 12876_2026_4663_MOESM5_ESM.zip › uncropped GEL/Suppl_Uncropped_WB_Fig3A _p-NF-κB p65_Repeat2.tif]

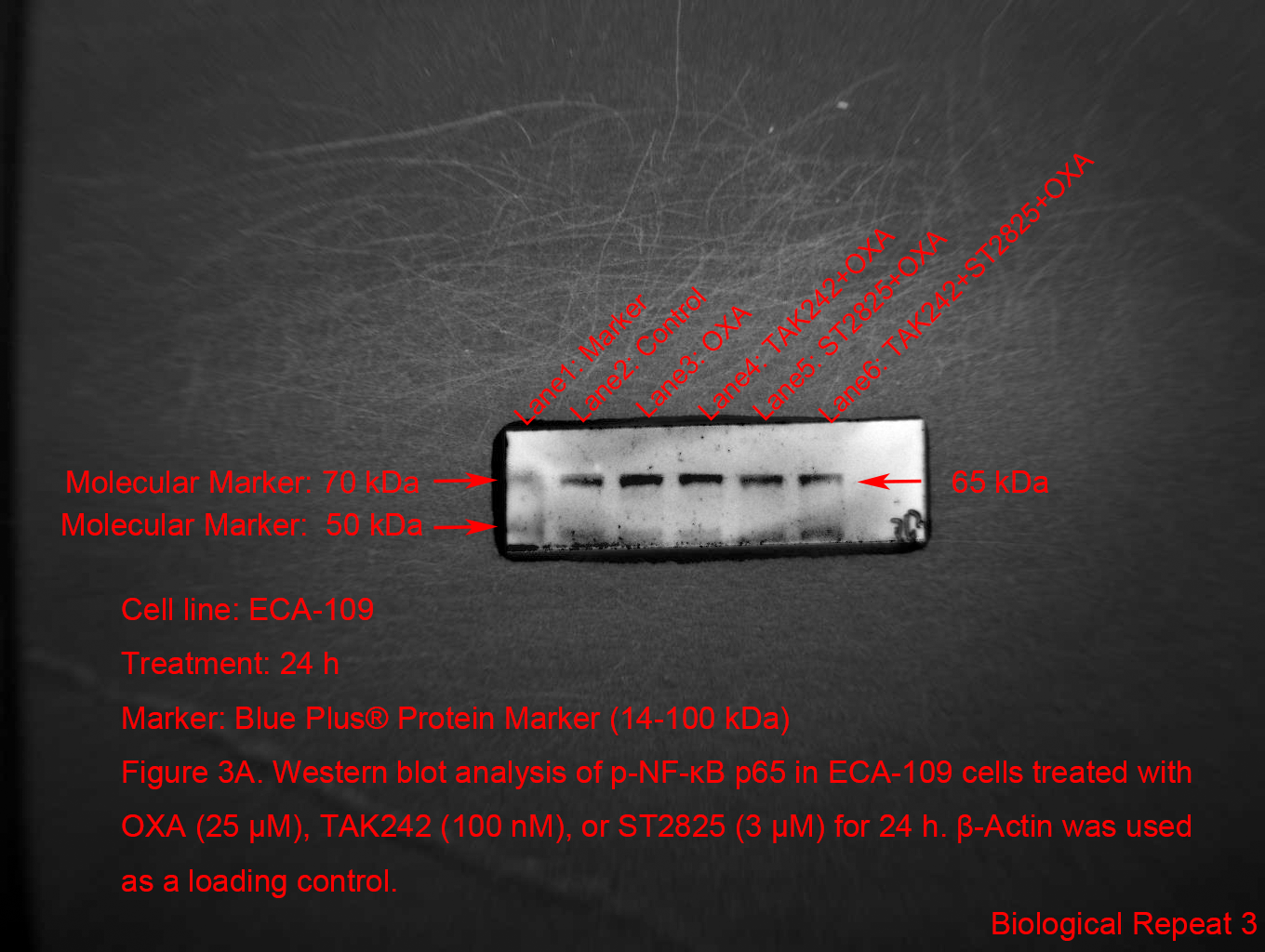

Supplement: Supplementary file 5 — Supplementary Material 5. [file 12876_2026_4663_MOESM5_ESM.zip › uncropped GEL/Suppl_Uncropped_WB_Fig3A _p-NF-κB p65_Repeat3.tif]

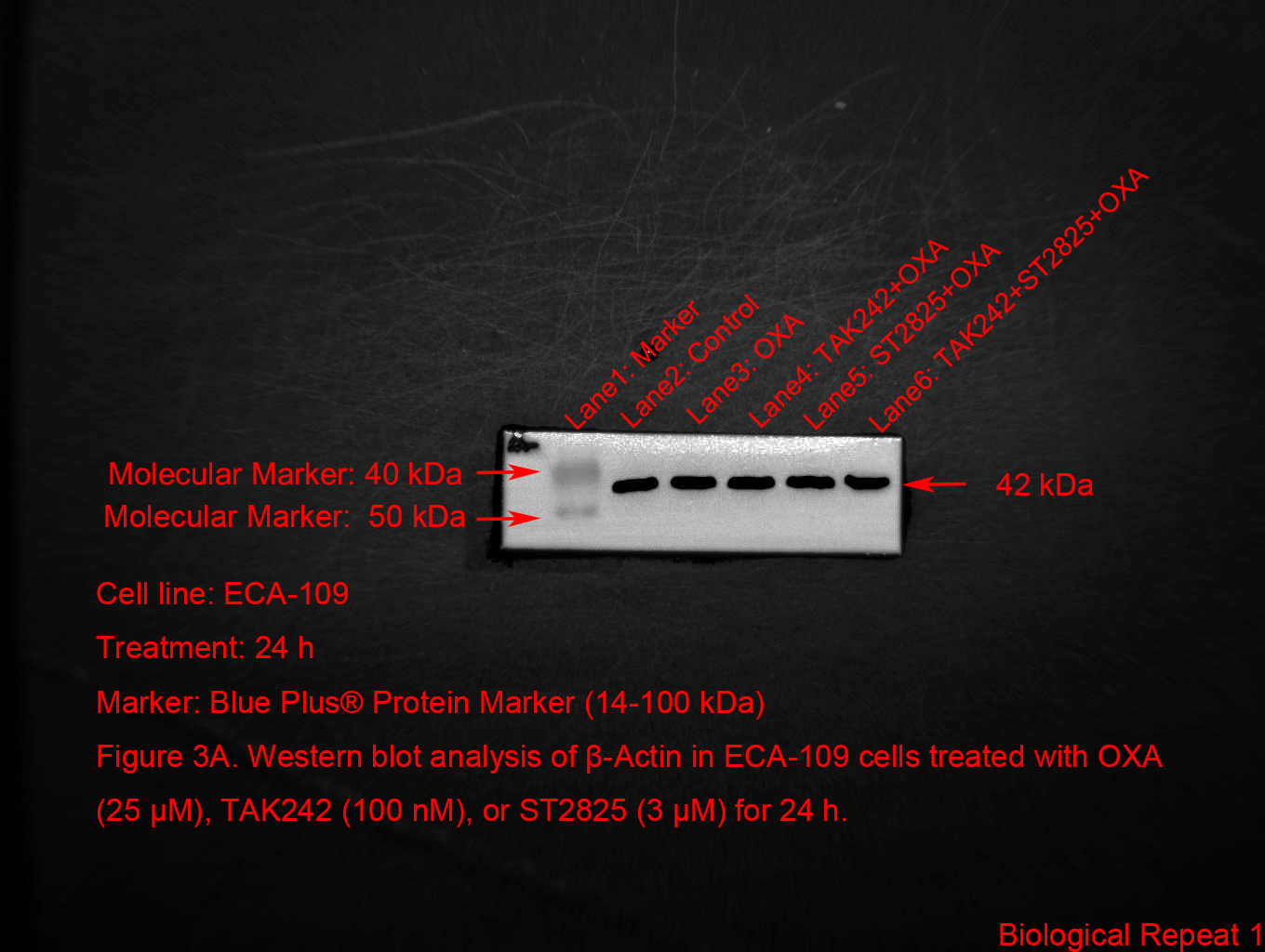

Supplement: Supplementary file 5 — Supplementary Material 5. [file 12876_2026_4663_MOESM5_ESM.zip › uncropped GEL/Suppl_Uncropped_WB_Fig3A _β-Actin_Repeat1.tif]

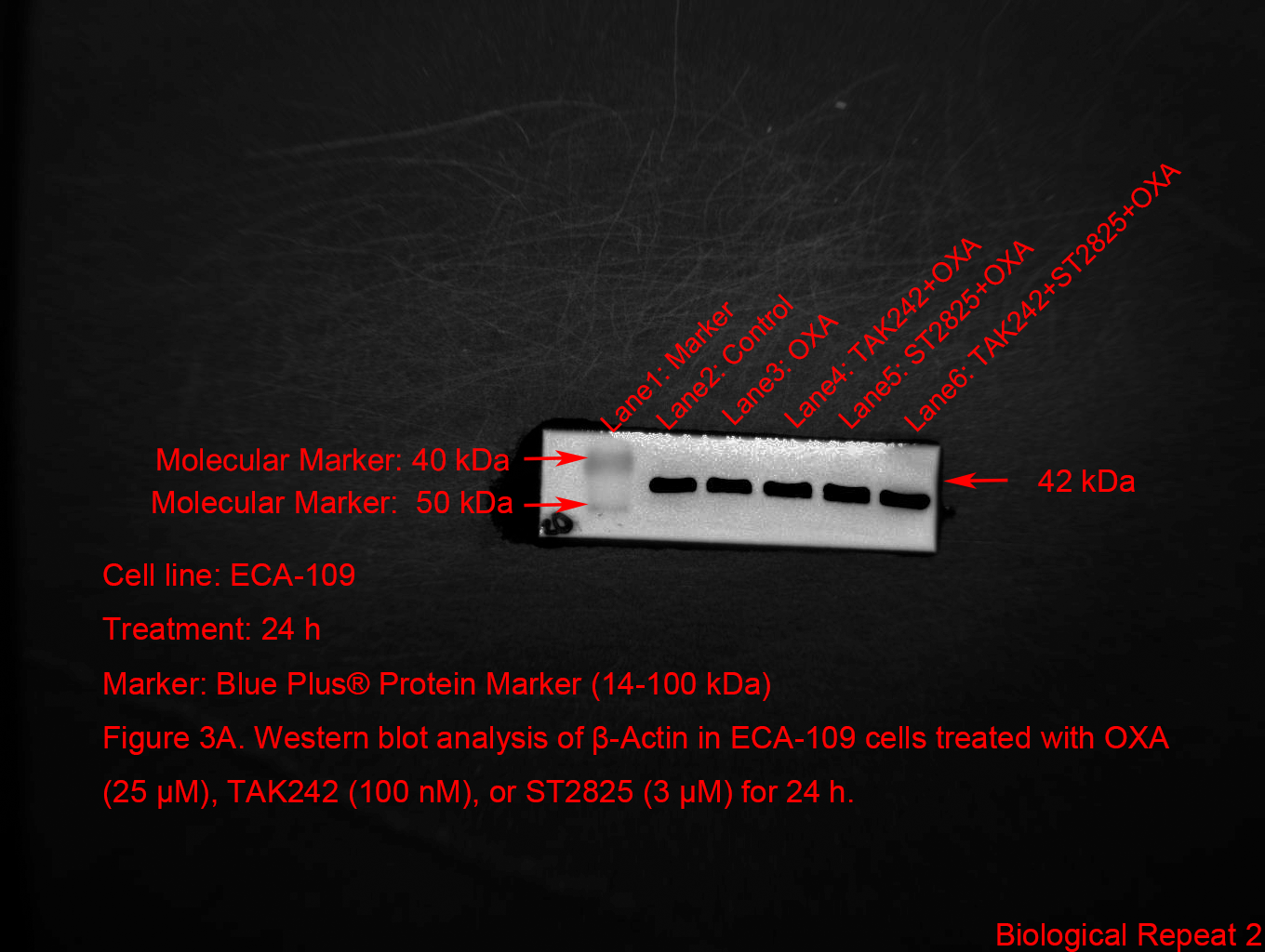

Supplement: Supplementary file 5 — Supplementary Material 5. [file 12876_2026_4663_MOESM5_ESM.zip › uncropped GEL/Suppl_Uncropped_WB_Fig3A _β-Actin_Repeat2.tif]

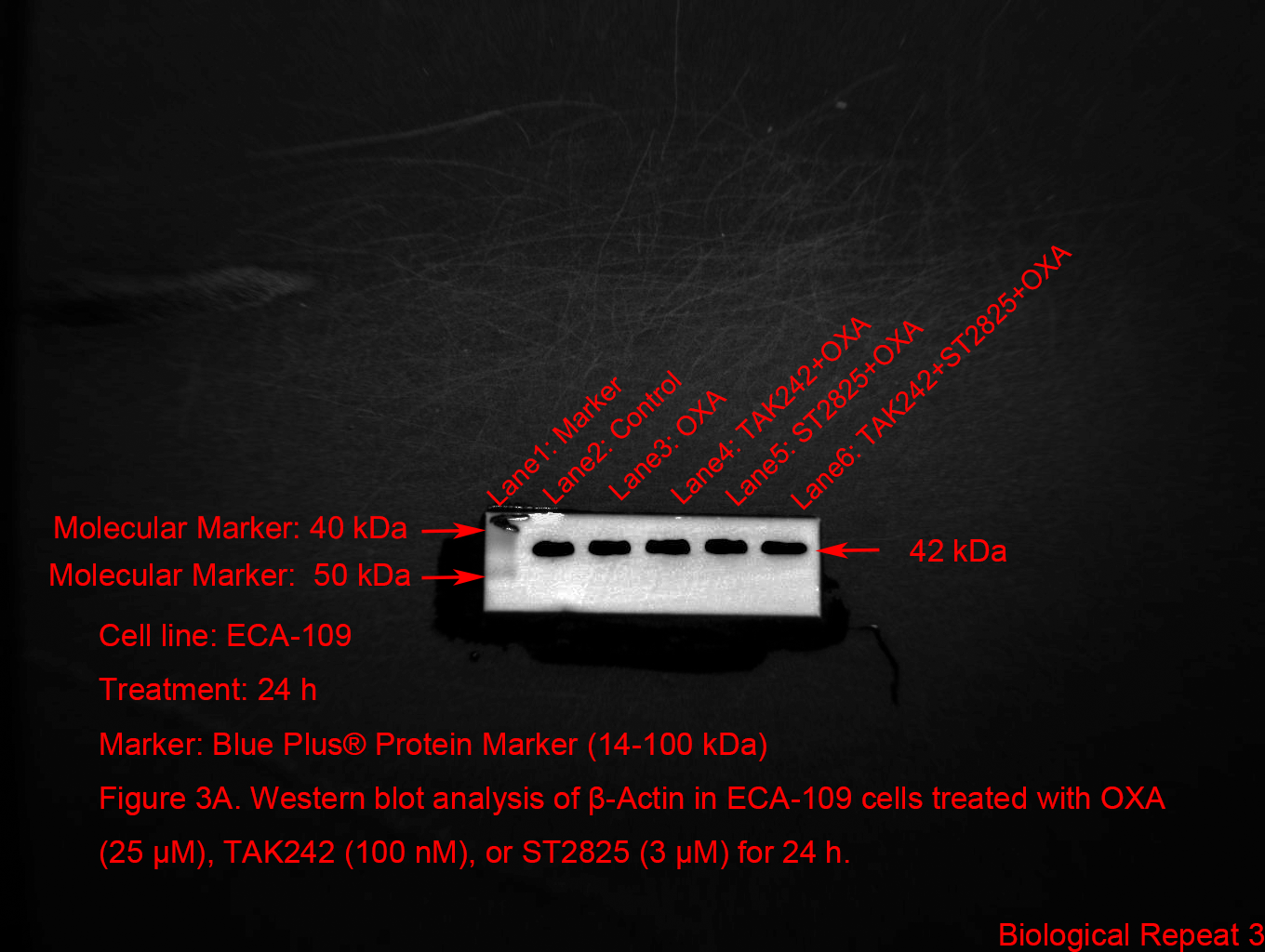

Supplement: Supplementary file 5 — Supplementary Material 5. [file 12876_2026_4663_MOESM5_ESM.zip › uncropped GEL/Suppl_Uncropped_WB_Fig3A _β-Actin_Repeat3.tif]

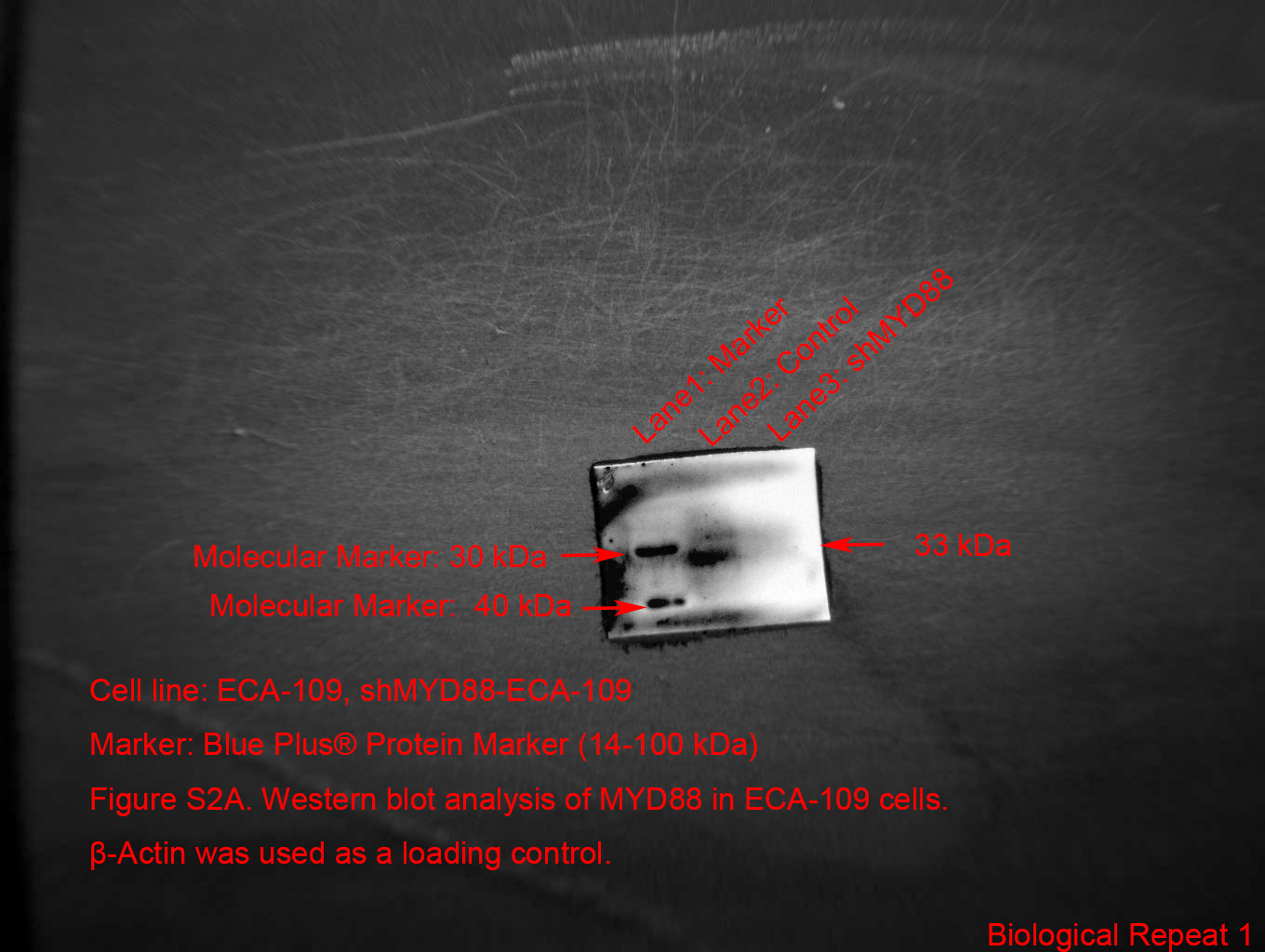

Supplement: Supplementary file 5 — Supplementary Material 5. [file 12876_2026_4663_MOESM5_ESM.zip › uncropped GEL/Suppl_Uncropped_WB_FigS2A _MYD88_Repeat1.tif]

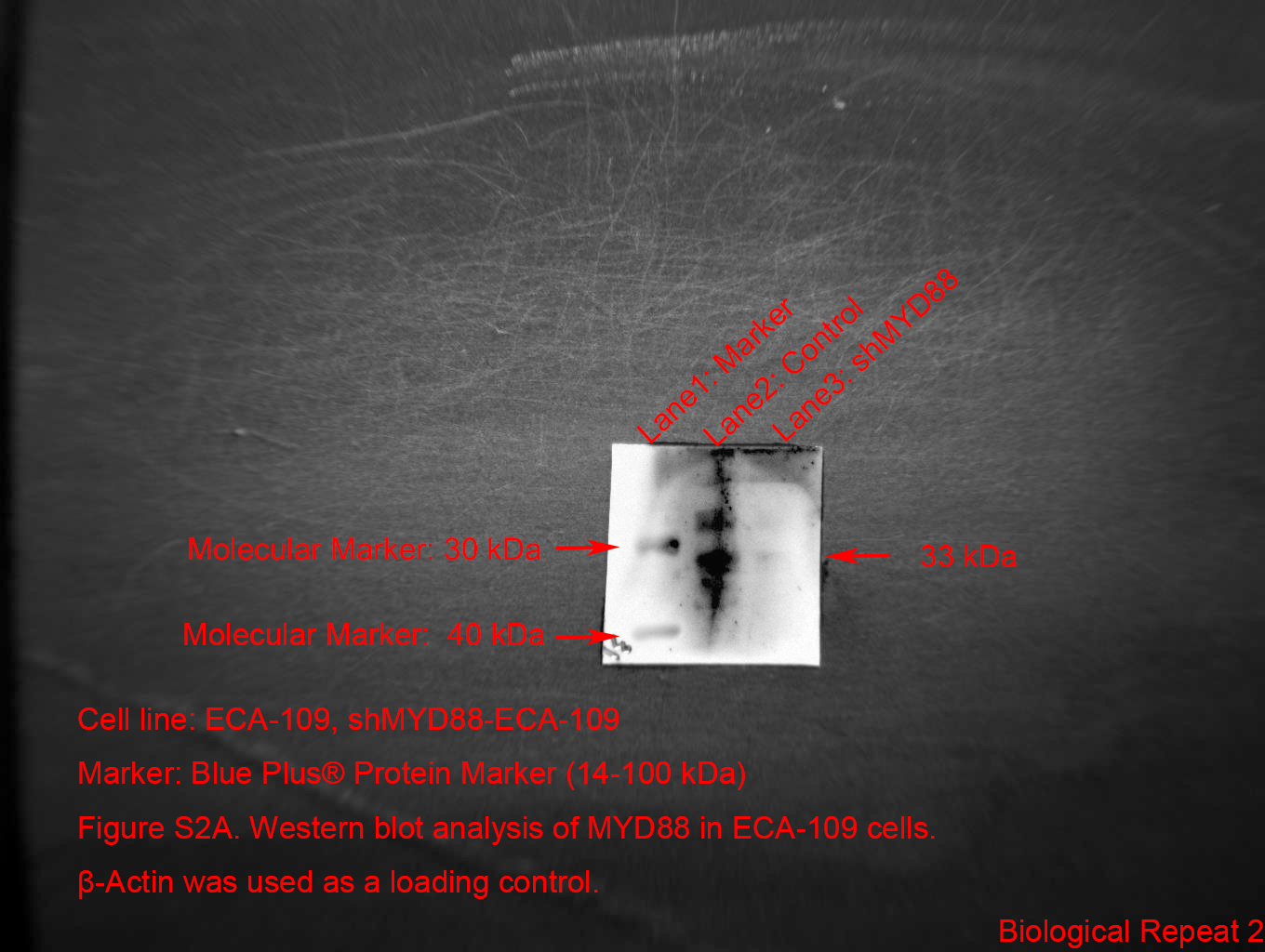

Supplement: Supplementary file 5 — Supplementary Material 5. [file 12876_2026_4663_MOESM5_ESM.zip › uncropped GEL/Suppl_Uncropped_WB_FigS2A _MYD88_Repeat2.tif]

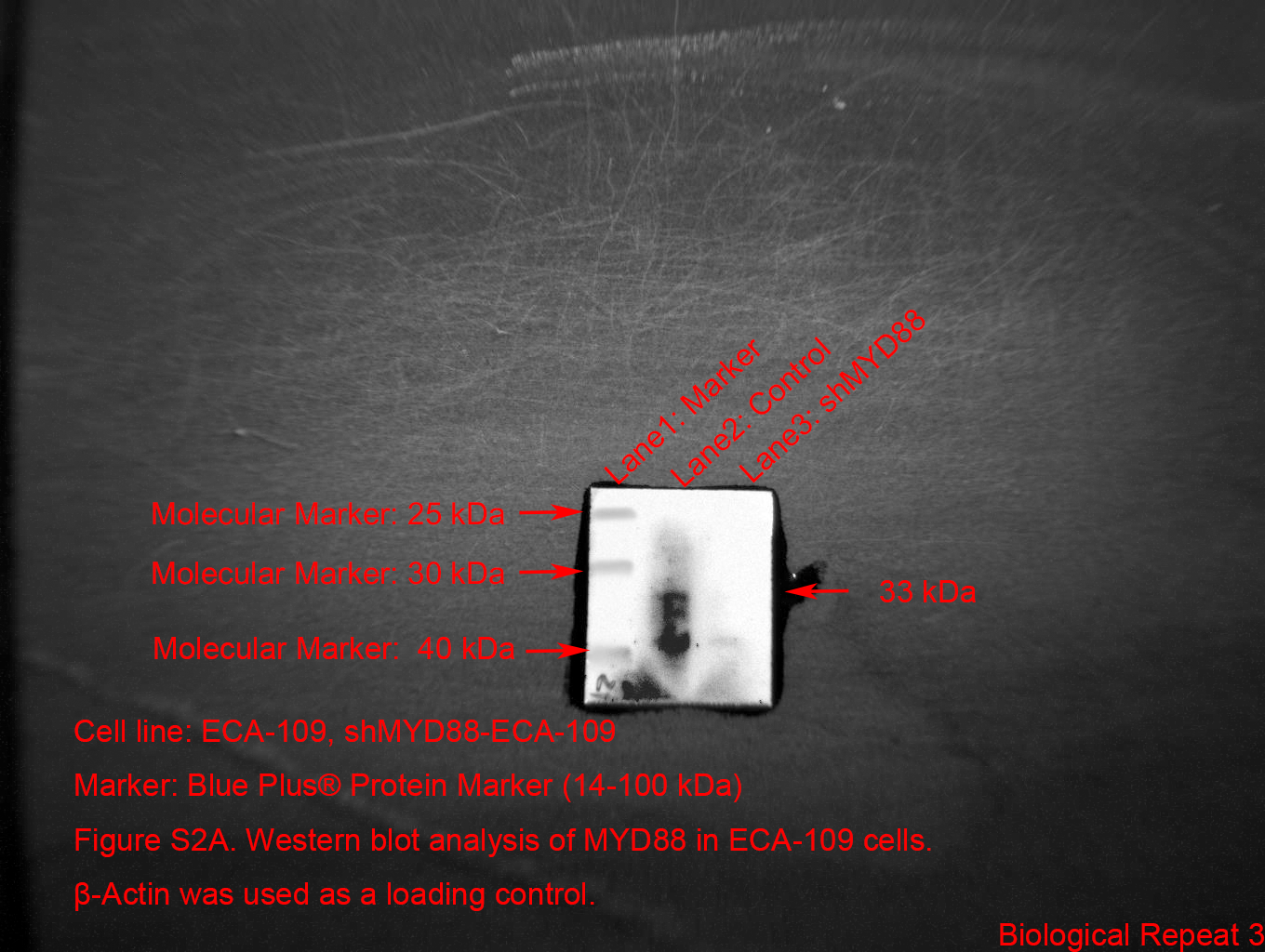

Supplement: Supplementary file 5 — Supplementary Material 5. [file 12876_2026_4663_MOESM5_ESM.zip › uncropped GEL/Suppl_Uncropped_WB_FigS2A _MYD88_Repeat3.tif]

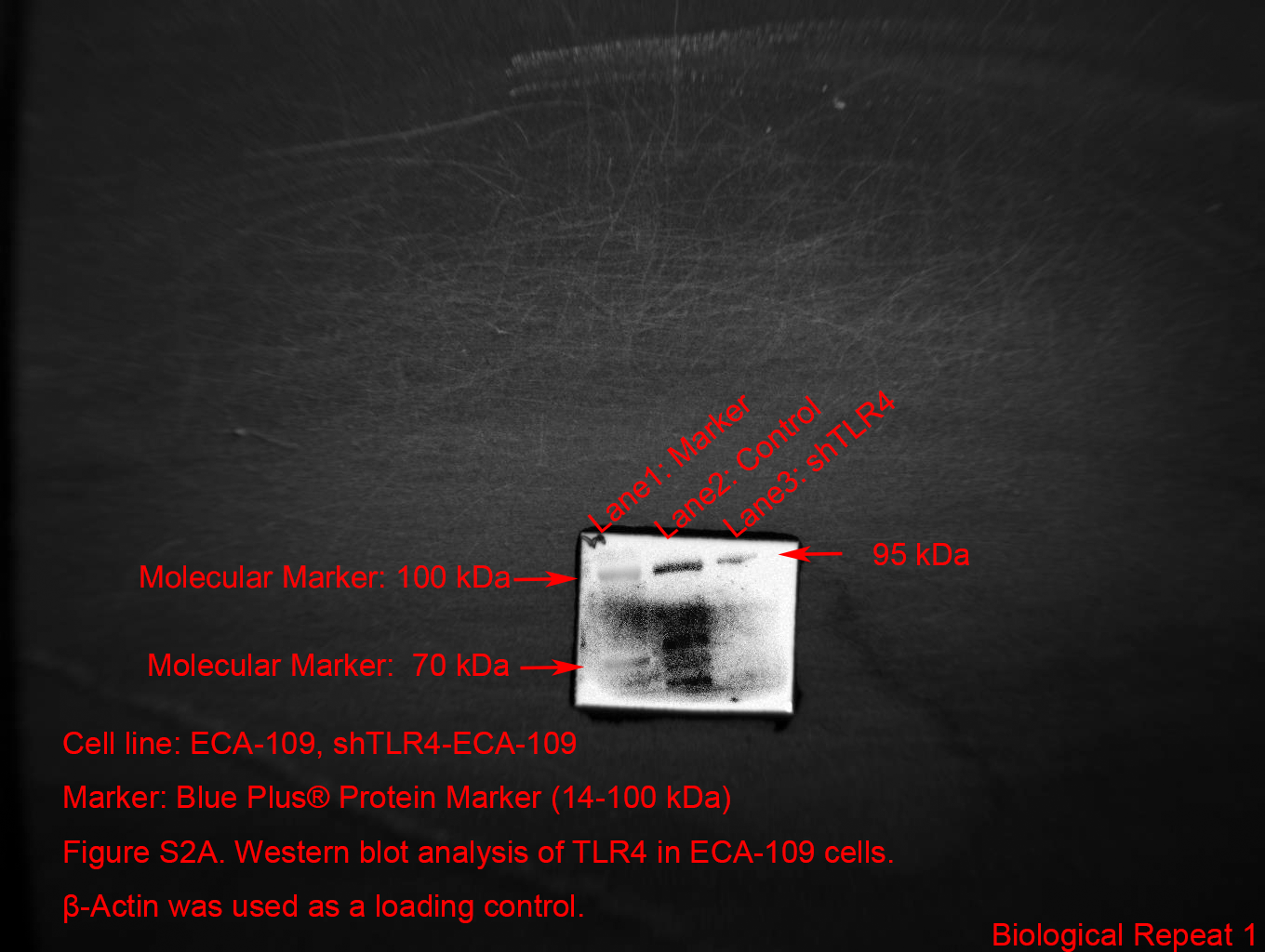

Supplement: Supplementary file 5 — Supplementary Material 5. [file 12876_2026_4663_MOESM5_ESM.zip › uncropped GEL/Suppl_Uncropped_WB_FigS2A _TLR4_Repeat1.tif]

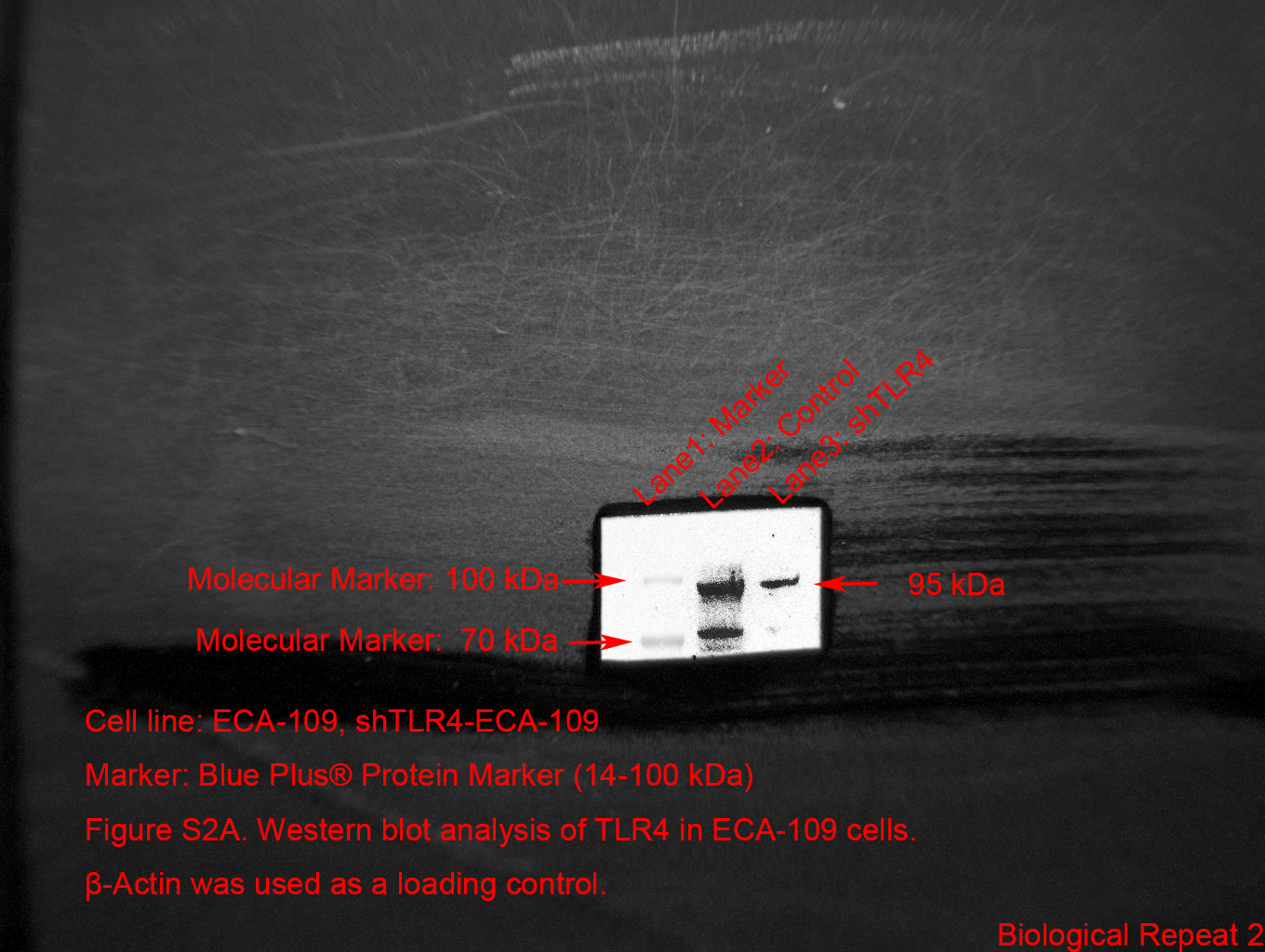

Supplement: Supplementary file 5 — Supplementary Material 5. [file 12876_2026_4663_MOESM5_ESM.zip › uncropped GEL/Suppl_Uncropped_WB_FigS2A _TLR4_Repeat2.tif]

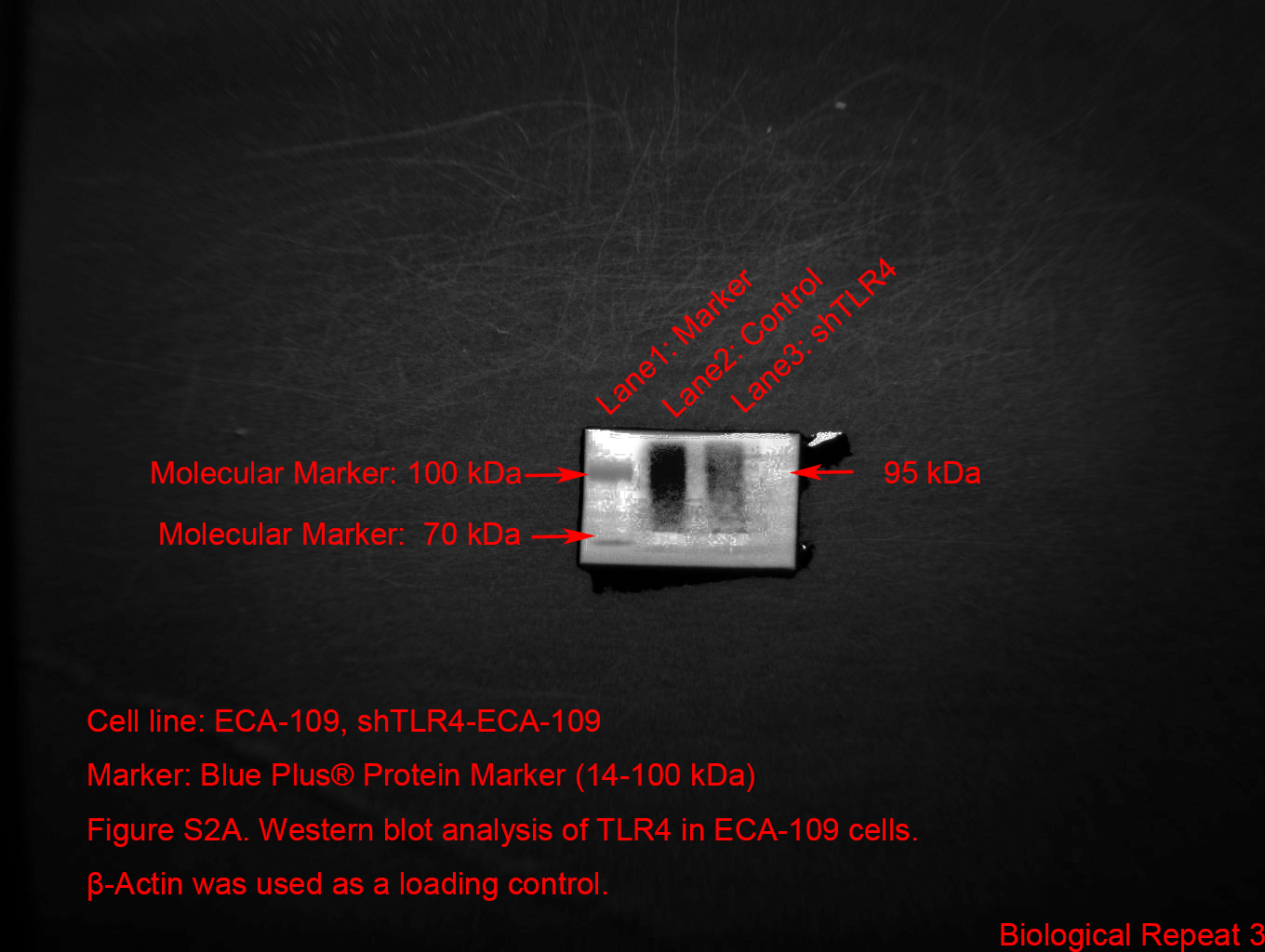

Supplement: Supplementary file 5 — Supplementary Material 5. [file 12876_2026_4663_MOESM5_ESM.zip › uncropped GEL/Suppl_Uncropped_WB_FigS2A _TLR4_Repeat3.tif]

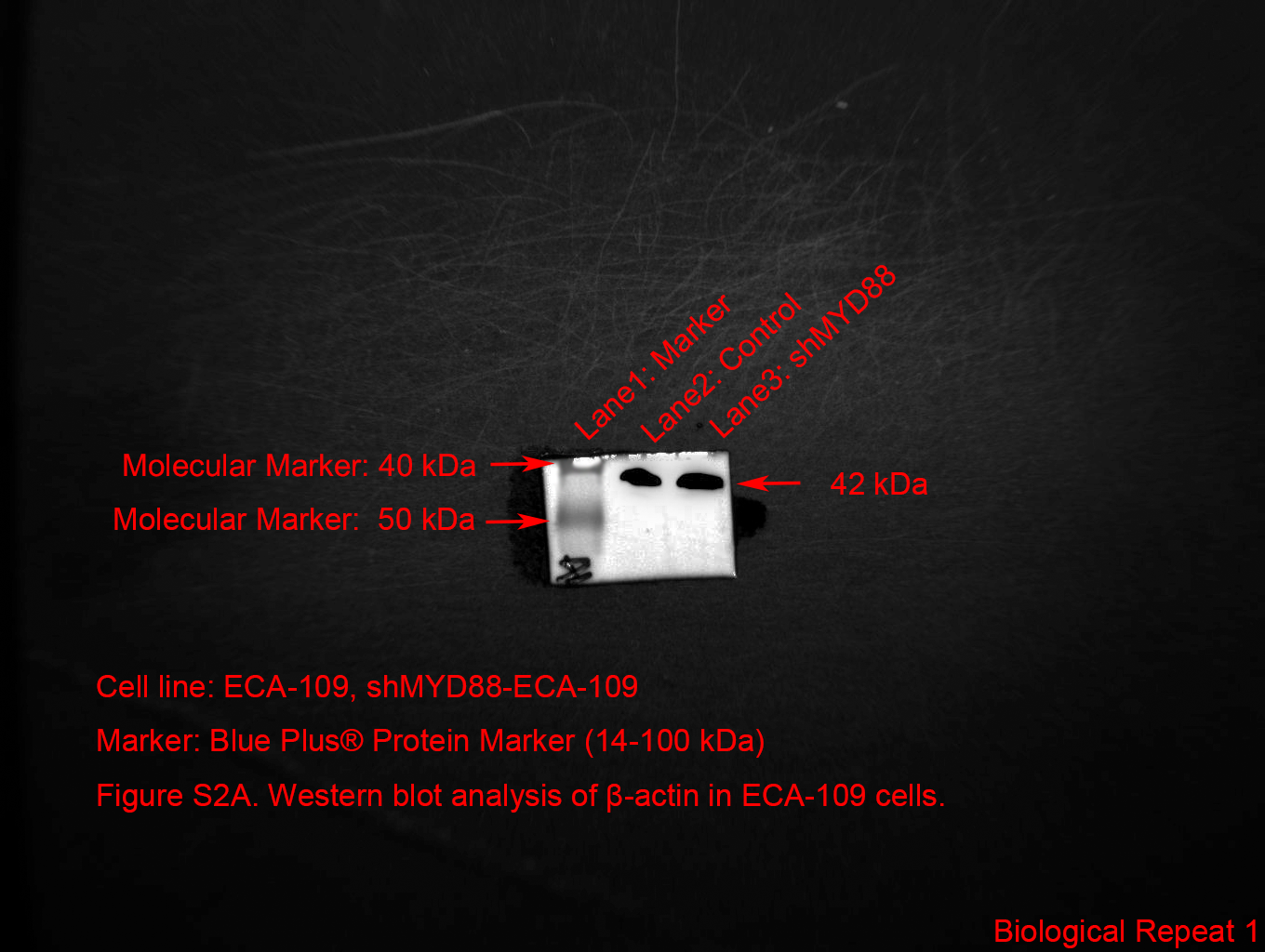

Supplement: Supplementary file 5 — Supplementary Material 5. [file 12876_2026_4663_MOESM5_ESM.zip › uncropped GEL/Suppl_Uncropped_WB_FigS2A _β-actin(shMYD88)_Repeat1.tif]

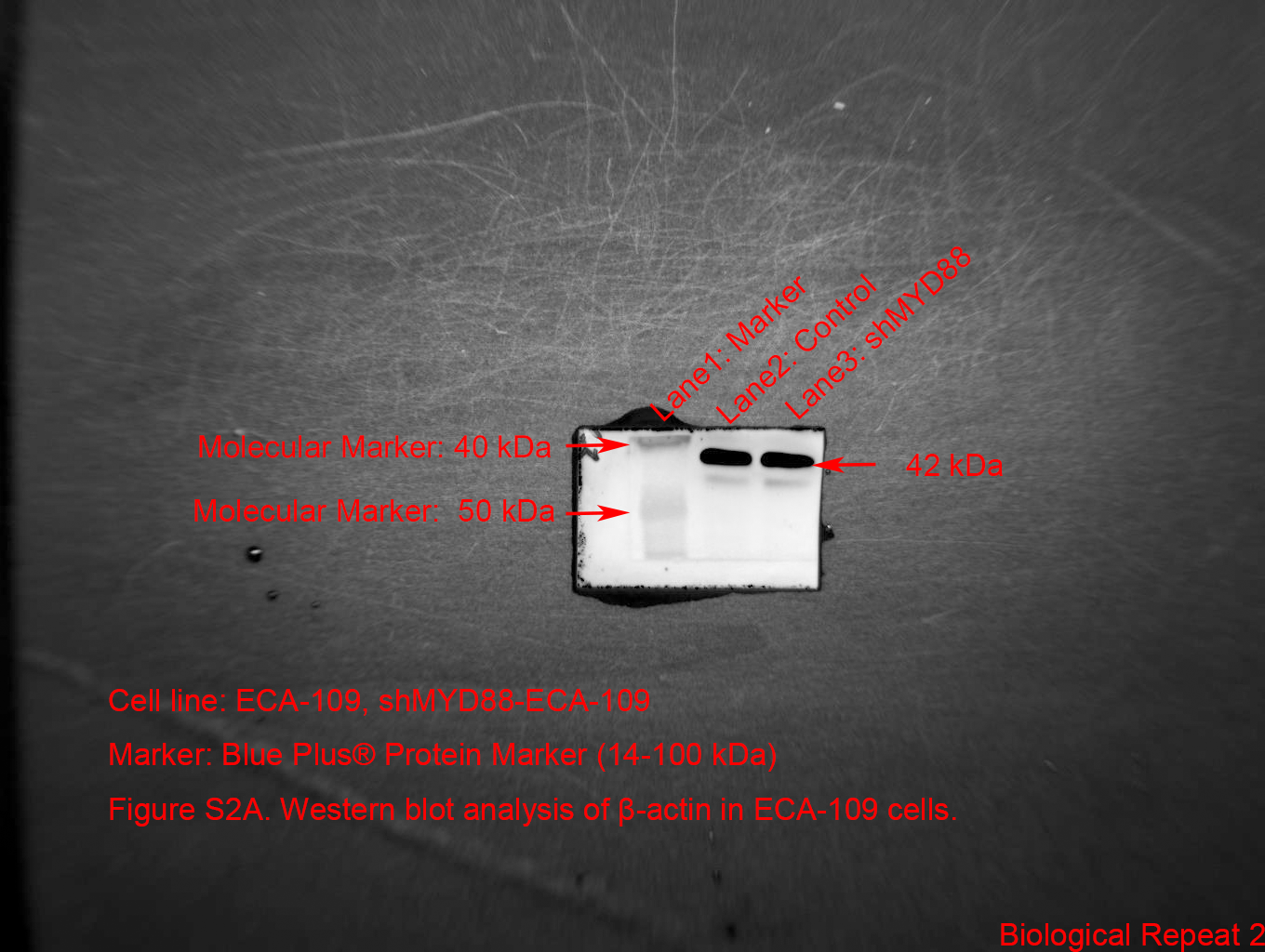

Supplement: Supplementary file 5 — Supplementary Material 5. [file 12876_2026_4663_MOESM5_ESM.zip › uncropped GEL/Suppl_Uncropped_WB_FigS2A _β-actin(shMYD88)_Repeat2.tif]

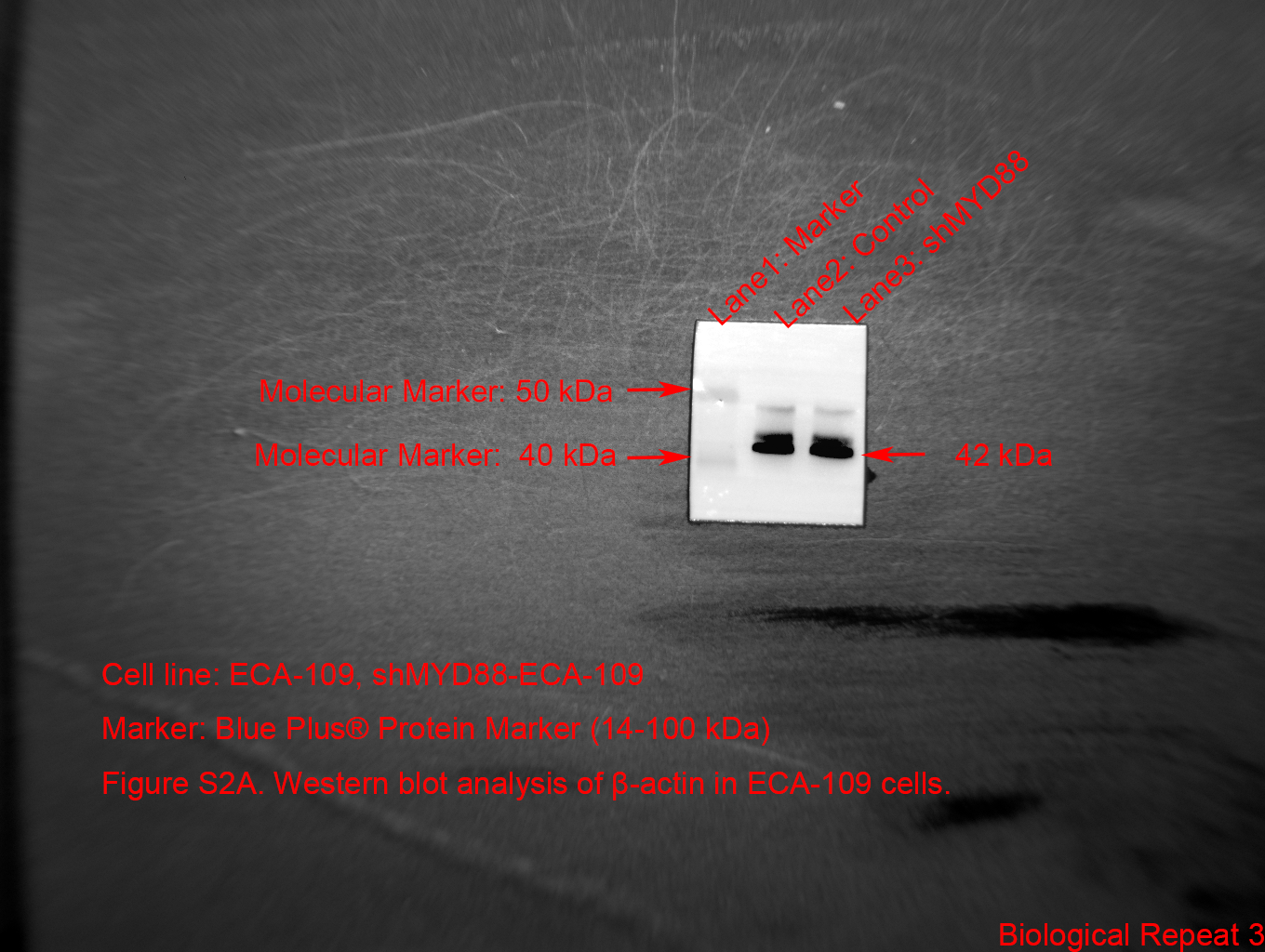

Supplement: Supplementary file 5 — Supplementary Material 5. [file 12876_2026_4663_MOESM5_ESM.zip › uncropped GEL/Suppl_Uncropped_WB_FigS2A _β-actin(shMYD88)_Repeat3.tif]

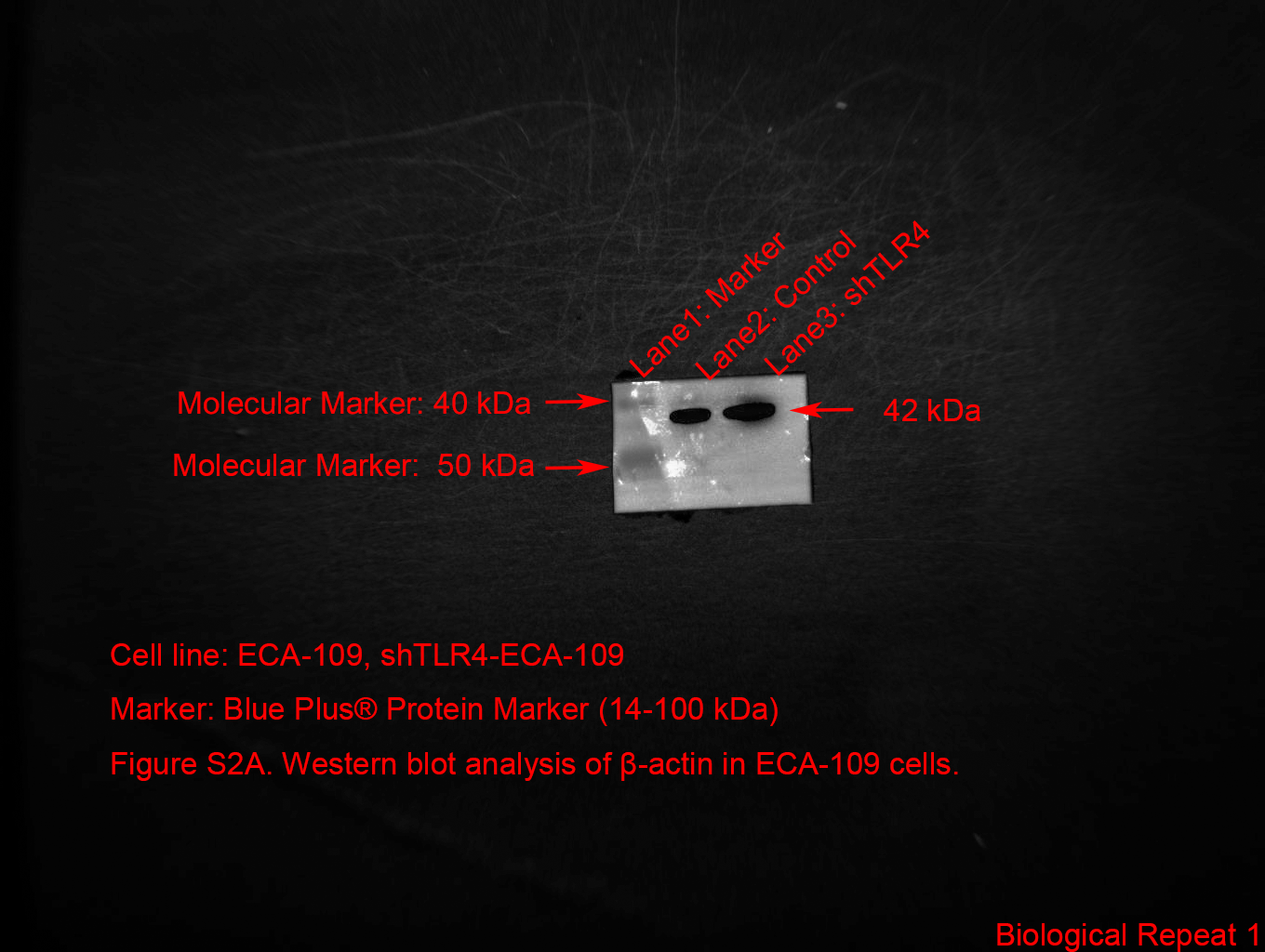

Supplement: Supplementary file 5 — Supplementary Material 5. [file 12876_2026_4663_MOESM5_ESM.zip › uncropped GEL/Suppl_Uncropped_WB_FigS2A _β-actin(shTLR4)_Repeat1.tif]

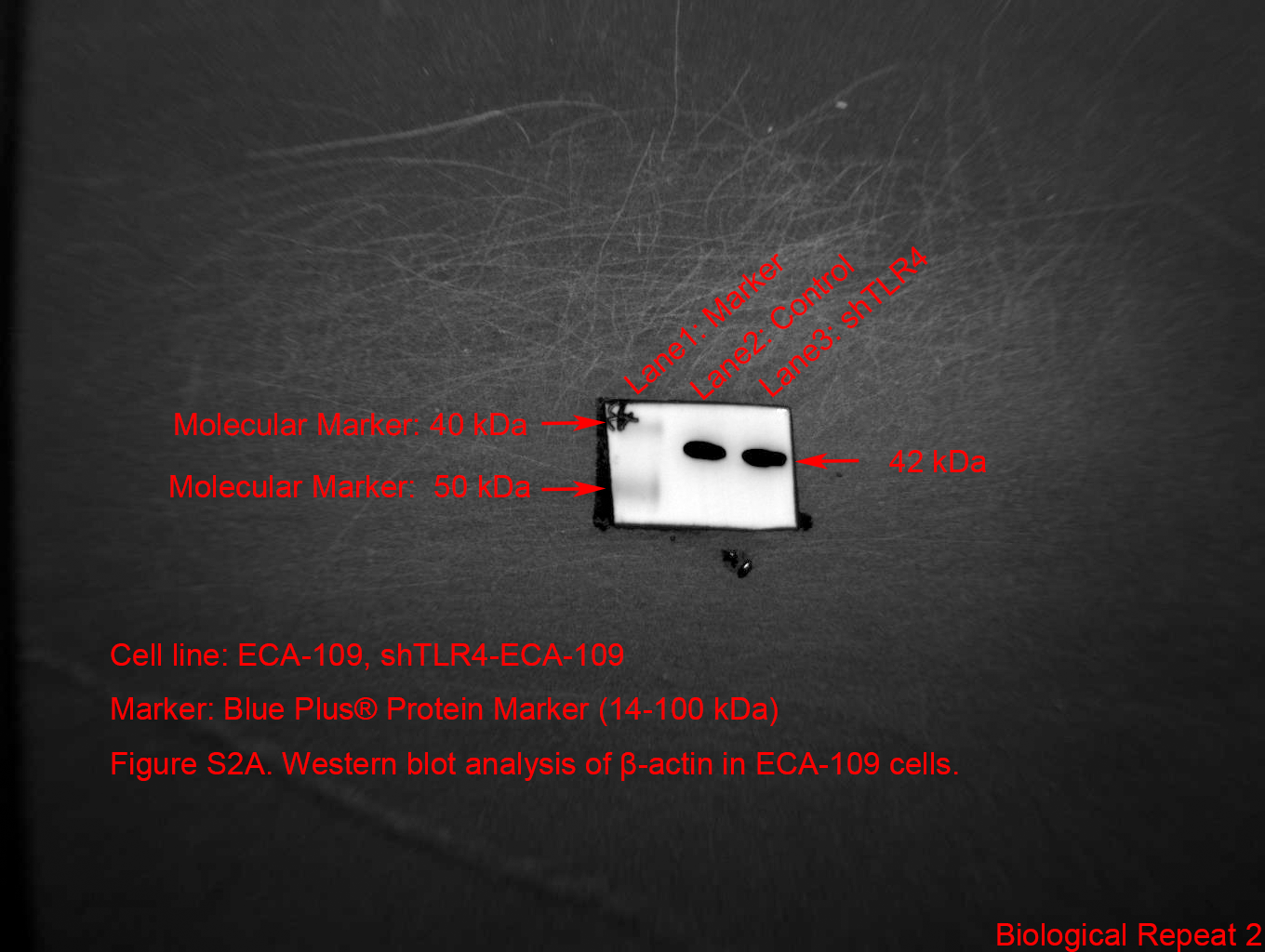

Supplement: Supplementary file 5 — Supplementary Material 5. [file 12876_2026_4663_MOESM5_ESM.zip › uncropped GEL/Suppl_Uncropped_WB_FigS2A _β-actin(shTLR4)_Repeat2.tif]

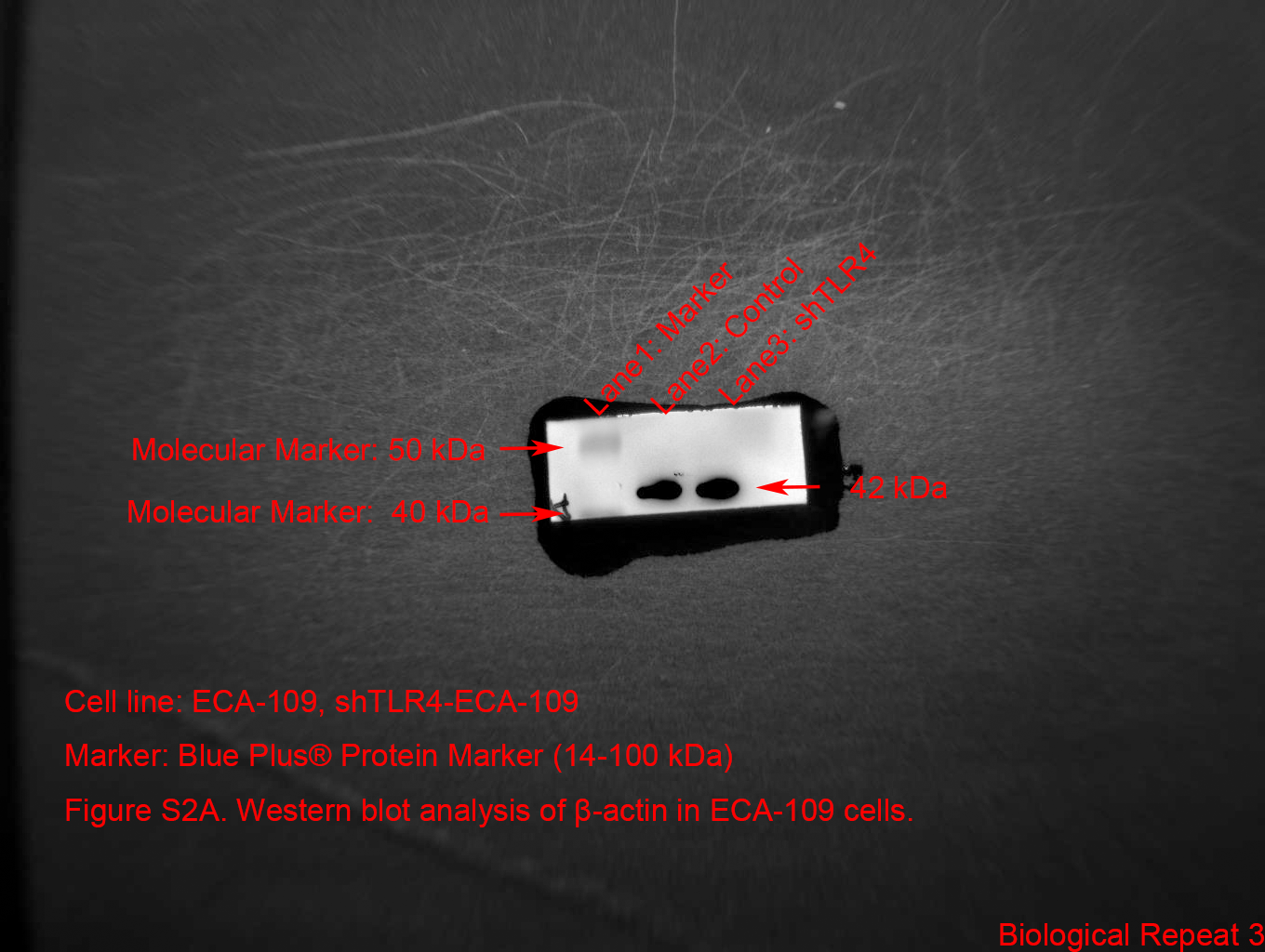

Supplement: Supplementary file 5 — Supplementary Material 5. [file 12876_2026_4663_MOESM5_ESM.zip › uncropped GEL/Suppl_Uncropped_WB_FigS2A _β-actin(shTLR4)_Repeat3.tif]
