# Supplementary material for: Inhibition of TLR4 enhances oxaliplatin chemotherapy sensitivity in esophageal squamous cell carcinoma by suppressing inflammation and glycolysis
Source: BMC Gastroenterol. 2026 Feb 10;26:168. doi: 10.1186/s12876-026-04663-2 (PMC12990394; doi:10.1186/s12876-026-04663-2)
Supplement: Supplementary file 5 — Supplementary Material 5. [file 12876_2026_4663_MOESM5_ESM.zip › uncropped GEL/Uncropped GELs.docx]

Supplementary File Legends for Inhibition of TLR4 Enhances Oxaliplatin Chemotherapy Sensitivity in Esophageal Squamous Cell Carcinoma by Suppressing Inflammation and Glycolysis

**Figure 3A**

**Suppl_Uncropped_WB_Fig3A_p-NF-κB p65_Repeat1.tif** Uncropped western blot image for phosphorylated NF-κB p65 (p-NF-κB p65) in ECA-109 cells from the same experiment as Figure 3A (biological repeat 1). Treatment conditions are consistent with the NF-κB p65 detection (Repeat 1), and p-NF-κB p65 was detected at 65 kDa.

**Suppl_Uncropped_WB_Fig3A_NF-κB p65_Repeat1.tif** Uncropped western blot image for NF-κB p65 in ECA-109 cells treated with OXA (25 μM), TAK242 (100 nM), or ST2825 (3 μM) for 24 h (biological repeat 1). Corresponding to Figure 3A in the main text. Run on a separate gel with consistent sample loading, run simultaneously, and identical experimental conditions as Suppl_Uncropped_WB_Fig3A_p-NF-κB p65_Repeat1.tif detection.

**Suppl_Uncropped_WB_Fig3A_β-Actin_Repeat1.tif** Uncropped western blot image for β-Actin (loading control, 42 kDa) in ECA-109 cells from the same experiment as Figure 3A (biological repeat 1). Run on a separate gel with consistent sample loading, run simultaneously, and identical experimental conditions as Suppl_Uncropped_WB_Fig3A_p-NF-κB p65_Repeat1.tif detection.

**Suppl_Uncropped_WB_Fig3A_p-NF-κB p65_Repeat2.tif** Uncropped western blot image for p-NF-κB p65 (65 kDa) in ECA-109 cells (biological repeat 2), corresponding to Figure 3A in the main text. Treatment conditions (OXA 25 μM, TAK242 100 nM, ST2825 3 μM, 24 h incubation) are consistent with Repeat 1.

**Suppl_Uncropped_WB_Fig3A_NF-κB p65_Repeat2.tif** Uncropped western blot image for NF-κB p65 (65 kDa) in ECA-109 cells (biological repeat 2), from the same experiment as p-NF-κB p65 (Repeat 2). Run on a separate gel with consistent sample loading, run simultaneously, and identical experimental conditions as Suppl_Uncropped_WB_Fig3A_p-NF-κB p65_Repeat2.tif detection.

**Suppl_Uncropped_WB_Fig3A_β-Actin_Repeat2.tif** Uncropped western blot image for β-Actin (loading control, 42 kDa) in ECA-109 cells (biological repeat 2), from the same experiment as Figure 3A. Run on a separate gel with consistent sample loading, run simultaneously, and identical experimental conditions as Suppl_Uncropped_WB_Fig3A_p-NF-κB p65_Repeat2.tif detection. Confirms equal sample loading with treatment conditions consistent with Repeats 1-2.

**Suppl_Uncropped_WB_Fig3A_p-NF-κB p65_Repeat3.tif** Uncropped western blot image for p-NF-κB p65 (65 kDa) in ECA-109 cells (biological repeat 3), corresponding to Figure 3A in the main text. Treatment conditions are identical to Repeats 1-2 (OXA 25 μM, TAK242 100 nM, ST2825 3 μM, 24 h incubation).

**Suppl_Uncropped_WB_Fig3A_NF-κB p65_Repeat3.tif** Uncropped western blot image for NF-κB p65 (65 kDa) in ECA-109 cells (biological repeat 3), from the same experiment as p-NF-κB p65 (Repeat 3). Run on a separate gel with consistent sample loading, run simultaneously, and identical experimental conditions as Suppl_Uncropped_WB_Fig3A_p-NF-κB p65_Repeat3.tif detection.

**Suppl_Uncropped_WB_Fig3A_β-Actin_Repeat3.tif** Uncropped western blot image for β-Actin (loading control, 42 kDa) in ECA-109 cells (biological repeat 3), from the same experiment as Figure 3A. Run on a separate gel with consistent sample loading, run simultaneously, and identical experimental conditions as Suppl_Uncropped_WB_Fig3A_p-NF-κB p65_Repeat3.tif detection. Ensures equal sample loading with consistent treatment conditions across all three biological repeats.

**Figure S2**

**Suppl_Uncropped_WB_FigS2A_TLR4_Repeat1.tif** Uncropped western blot image for TLR4 (molecular weight ~95 kDa) in ECA-109 cells transfected with shTLR4 (TLR4 knockdown) or shNC (negative control) (biological repeat 1). TLR4 expression was detected to verify knockdown efficiency, with treatment conditions consistent across groups.

**Suppl_Uncropped_WB_FigS2A_β-actin(shTLR4)_Repeat1.tif** Uncropped western blot image for β-Actin (loading control, 42 kDa) in ECA-109 cells from the same experiment as TLR4 detection (shTLR4 vs shNC, repeat 1). Run on a separate gel with consistent sample loading, run simultaneously, and identical experimental conditions as Suppl_Uncropped_WB_FigS2A_TLR4_Repeat1.tif detection. Ensures equal sample loading across groups.

**Suppl_Uncropped_WB_FigS2A_MYD88_Repeat1.tif** Uncropped western blot image for MYD88 (~33 kDa) in ECA-109 cells transfected with shMYD88 (MYD88 knockdown) or shNC (biological repeat 1). MYD88 expression was analyzed to confirm knockdown efficiency, with experimental conditions matching the TLR4 detection assay.

**Suppl_Uncropped_WB_FigS2A_β-actin(shMYD88)_Repeat1.tif** Uncropped western blot image for β-Actin (loading control, 42 kDa) in ECA-109 cells from the same experiment as MYD88 detection (shMYD88 vs shNC, repeat 1). Run on a separate gel with consistent sample loading, run simultaneously, and identical experimental conditions as Suppl_Uncropped_WB_FigS2A_MYD88_Repeat1.tif detection. Verifies consistent sample loading.

**Suppl_Uncropped_WB_FigS2A_TLR4_Repeat2.tif** Uncropped western blot image for TLR4 (molecular weight ~95 kDa) in ECA-109 cells transfected with shTLR4 (TLR4 knockdown) or shNC (negative control) (biological repeat 2). TLR4 expression was detected to verify knockdown efficiency, with treatment conditions consistent with Repeat 1.

**Suppl_Uncropped_WB_FigS2A_β-actin(shTLR4)_Repeat2.tif** Uncropped western blot image for β-Actin (loading control, 42 kDa) in ECA-109 cells from the same experiment as TLR4 detection (shTLR4 vs shNC, repeat 2). Run on a separate gel with consistent sample loading, run simultaneously, and identical experimental conditions as Suppl_Uncropped_WB_FigS2A_TLR4_Repeat2.tif detection. Ensures equal sample loading across groups.

**Suppl_Uncropped_WB_FigS2A_MYD88_Repeat2.tif** Uncropped western blot image for MYD88 (~33 kDa) in ECA-109 cells transfected with shMYD88 (MYD88 knockdown) or shNC (biological repeat 2). MYD88 expression was analyzed to confirm knockdown efficiency, with experimental conditions matching the TLR4 detection assay (Repeat 2).

**Suppl_Uncropped_WB_FigS2A_β-actin(shMYD88)_Repeat2.tif** Uncropped western blot image for β-Actin (loading control, 42 kDa) in ECA-109 cells from the same experiment as MYD88 detection (shMYD88 vs shNC, repeat 2). Run on a separate gel with consistent sample loading, run simultaneously, and identical experimental conditions as Suppl_Uncropped_WB_FigS2A_MYD88_Repeat2.tif detection. Verifies consistent sample loading.

**Suppl_Uncropped_WB_FigS2A_TLR4_Repeat3.tif** Uncropped western blot image for TLR4 (molecular weight ~95 kDa) in ECA-109 cells transfected with shTLR4 (TLR4 knockdown) or shNC (negative control) (biological repeat 3). TLR4 expression was detected to verify knockdown efficiency, with treatment conditions consistent with Repeats 1-2.

**Suppl_Uncropped_WB_FigS2A_β-actin(shTLR4)_Repeat3.tif** Uncropped western blot image for β-Actin (loading control, 42 kDa) in ECA-109 cells from the same experiment as TLR4 detection (shTLR4 vs shNC, repeat 3). Run on a separate gel with consistent sample loading, run simultaneously, and identical experimental conditions as Suppl_Uncropped_WB_FigS2A_TLR4_Repeat3.tif detection. Ensures equal sample loading across groups.

**Suppl_Uncropped_WB_FigS2A_MYD88_Repeat3.tif** Uncropped western blot image for MYD88 (~33 kDa) in ECA-109 cells transfected with shMYD88 (MYD88 knockdown) or shNC (biological repeat 3). MYD88 expression was analyzed to confirm knockdown efficiency, with experimental conditions matching the TLR4 detection assay (Repeats 1-2).

**Suppl_Uncropped_WB_FigS2A_β-actin(shMYD88)_Repeat3.tif** Uncropped western blot image for β-Actin (loading control, 42 kDa) in ECA-109 cells from the same experiment as MYD88 detection (shMYD88 vs shNC, repeat 3). Run on a separate gel with consistent sample loading, run simultaneously, and identical experimental conditions as Suppl_Uncropped_WB_FigS2A_MYD88_Repeat3.tif detection. Verifies consistent sample loading.
